# Fine-tuning alkyne cycloadditions: Insights into photochemistry responsible for the double-strand DNA cleavage via structural perturbations in diaryl alkyne conjugates

**DOI:** 10.3762/bjoc.7.93

**Published:** 2011-06-16

**Authors:** Wang-Yong Yang, Samantha A Marrone, Nalisha Minors, Diego A R Zorio, Igor V Alabugin

**Affiliations:** 1 Department of Chemistry and Biochemistry, Florida State University, Tallahassee, FL 32306-4390, USA

**Keywords:** cancer cell proliferation assay, DNA alkylation, lysine conjugate, photocycloaddition, photo-DNA cleavage, plasmid relaxation assay, triplet excitation

## Abstract

Hybrid molecules combining photoactivated aryl acetylenes and a dicationic lysine moiety cause the most efficient double-strand (ds) DNA cleavage known to date for a small molecule. In order to test the connection between the alkylating ability and the DNA-damaging properties of these compounds, we investigated the photoreactivity of three isomeric aryl–tetrafluoropyridinyl (TFP) alkynes with amide substituents in different positions (*o*-, *m*-, and *p*-) toward a model π-system. Reactions with 1,4-cyclohexadiene (1,4-CHD) were used to probe the alkylating properties of the triplet excited states in these three isomers whilst Stern–Volmer quenching experiments were used to investigate the kinetics of photoinduced electron transfer (PET). The three analogous isomeric lysine conjugates cleaved DNA with different efficiencies (34, 15, and 0% of ds DNA cleavage for *p*-, *m*-, and *o*-substituted lysine conjugates, respectively) consistent with the alkylating ability of the respective acetamides. The significant protecting effect of the hydroxyl radical and singlet oxygen scavengers to DNA cleavage was shown only with *m*-lysine conjugate. All three isomeric lysine conjugates inhibited human melanoma cell growth under photoactivation: The *p*-conjugate had the lowest CC_50_ (50% cell cytotoxicity) value of 1.49 × 10^−7^ M.

## Introduction

Triggering chemical processes with light offers numerous practical advantages. Not only does photochemistry open an additional dimension for the control of chemical reactivity by enabling many, otherwise impossible, synthetic transformations, but this mode of activation also provides useful spatial and temporal control of chemical processes that are required to occur in the right place and at the right time. Such selectivity is particularly useful in biological applications such as cancer therapy where it accounts for the increasing importance of photodynamic therapy and related methods [1–11]. Previously, we expanded our studies of alkyne reactivity [12–23] to the design of photoactivated DNA cleavers, which combine a DNA-damaging part derived from diaryl alkynes and benzannelated enediynes with a cationic DNA-binding moiety.

**Figure 1:**
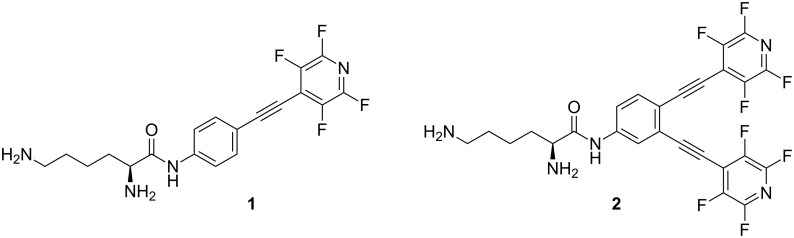
Structure of C-lysine conjugates.

The hybrid molecules that combined photoactivated alkynes with a dicationic moiety derived from lysine (C-lysine conjugates in Figure 1) displayed a combination of unique properties such as the ability to cause true double-strand (ds) DNA cleavage [24], amplification of ds cleavage dramatically at the lower pH of cancer cells [25], as well as the ability to recognize terminal phosphate monoester groups at the site of initial single-strand (ss) DNA damage and convert it into the more therapeutically important ds DNA damage [26].

We have shown that these compounds also could break intercellular DNA [27] and induce >90% cancer cell death at concentrations as low as 10 nM [25]. In spite of these remarkable properties, the mechanism of DNA cleavage by photoactivated alkynes and enediynes is still not fully understood.

Some light has been shed on the mechanism by the sequence selectivity of DNA cleavage in internally labeled DNA oligomers [28]. All enediyne-, alkyne-, and fulvene-based lysine conjugates displayed G-selective cleavage, especially at GG and GGG sites adjacent to the AT-rich sequence (the AT-tract), the preferred binding location for protonated amines. The G-selectivity is typical for oxidative DNA damage via PET for the most easily oxidized base, guanine. However, a noticeable amount of cleavage at a single G site in the AT-rich region is not consistent with purely oxidative DNA damage in the presence of spatially close GG and GGG sites, both of which are better sinks for the transient hole in the DNA. This observation suggests the presence of competitive DNA-cleavage mechanisms, such as guanine alkylation [29–35], which combine with the oxidative DNA damage to account for the efficient ds cleavage of plasmid DNA.

**Figure 2:**
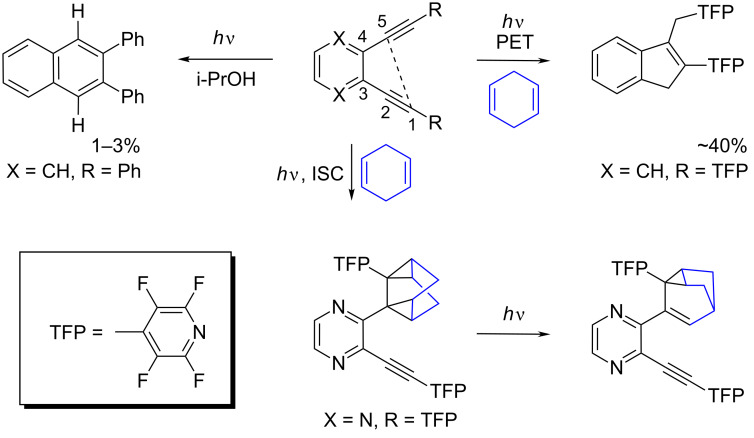
Alternative pathways of enediyne photoreactivity: photo-Bergman cyclization (left), C1–C5 cyclization (right), and triplet photocycloaddition (bottom). TFP = tetrafluoropyridinyl.

In the case of enediyne conjugate **2**, the additional DNA-cleavage mechanism may be provided by either photo-Bergman cyclization [3,36–44] (akin to such well-known DNA cleavers as enediyne antibiotics) [45–46] or C1–C5 cyclization [47–52] (Figure 2). In the latter process, which transforms enediynes into indenes, four hydrogens are transferred from the environment (two as H-atoms and two as protons), and thus DNA can be damaged via H-atom abstraction in a particularly efficient manner.

**Figure 3:**
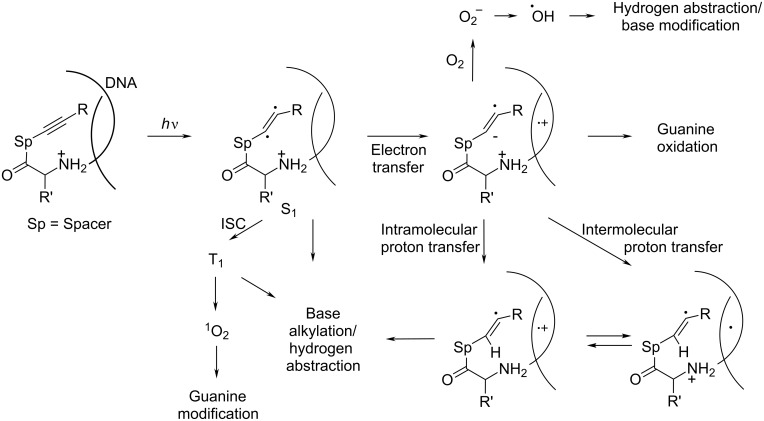
Summary of possible mechanistic alternatives for the observed DNA cleavage by monoacetylene conjugate **1**.

Efficient DNA cleavage by the monoacetylene conjugate **1**, which is capable of neither Bergman nor C1–C5 cyclization, suggests that other scenarios are possible and a more detailed understanding of alkyne photochemistry is vital for unraveling the mechanistic scenarios that account for DNA cleavage by these compounds (Figure 3) [25].

As illustrated in Figure 3, multiple reaction pathways are potentially unlocked by the photoactivation of alkyne conjugates. In the past, we observed dramatic differences in reactivity as a result of structural perturbations in the aryl moiety of diaryl alkynes. For example, introduction of strongly acceptor TFP substituents at the alkyne terminus changed the cyclization direction from the photo-Bergman closure to the C1–C5 cyclization due to the change in the nature of the key photophysical step and the involvement of PET from 1,4-cyclohexadiene (1,4-CHD) to the enediyne excited singlet state. In contrast, substituents that accelerate the intersystem crossing (ISC) through a “phantom state” effect [53–55] direct reactivity along an alternative triplet cycloaddition pathway.

Our previous mechanistic studies suggested that neither singlet oxygen nor diffusing oxygen- and carbon-centered radical species play a significant role under the conditions where the most efficient ds cleavage by monoalkynes is observed (pH 6) [25]. From the narrowed list of mechanistic scenarios, base alkylation remains a likely origin of the photodamaging ability of such alkynes. Such reactivity is consistent with the above-mentioned ability of alkynes to act as electrophilic alkylating agents toward electron-rich π-systems observed in triplet photocycloaddition of TFP-substituted diaryl acetylenes [53].

**Scheme 1:**
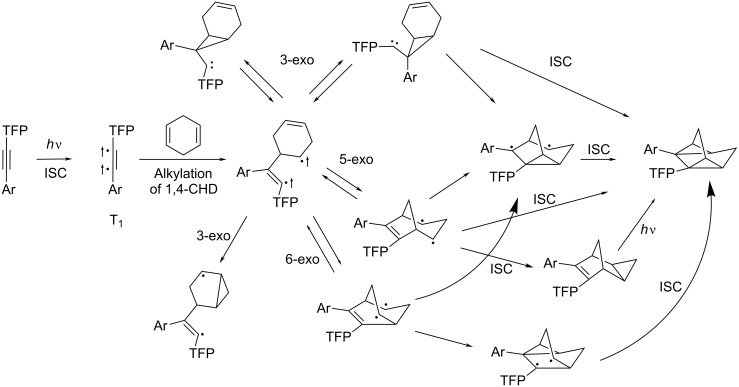
Proposed mechanism of photocycloaddition of acetylene with 1,4-CHD.

The mechanism of triplet photocycloaddition involves a sequence of radical closures initiated by the formation of a triplet 1,4-diradical via the reaction of 1,4-CHD and the alkyne π,π*-triplet state. Although several plausible mechanistic pathways converge at the same homoquadricyclane product in Scheme 1, the maximum quantum yield of 0.50 along with the DFT activation barriers at the triplet hypersurface suggest that 5-exo-trig attack of electrophilic vinyl radical at the remaining 1,4-CHD double bond is the most likely subsequent step.

Because this photocycloaddition occurs from the triplet state, the competition between triplet and singlet-state reactivity is likely to be important for the specifics of DNA photodamage. In particular, this competition would control the relative importance of PET which, in the case of moderately efficient electron donors, is only energetically favorable from the singlet excited state. The relative contribution of these two pathways should be reflected in two different mechanisms of DNA damage, i.e., oxidative DNA cleavage versus DNA alkylation.

**Figure 4:**
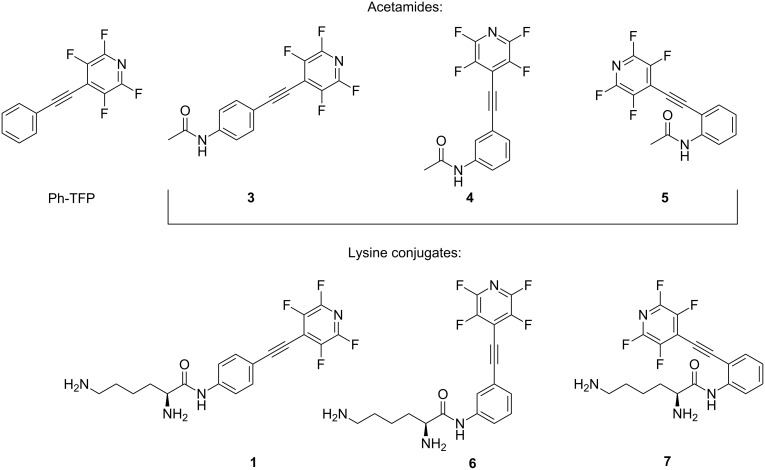
*p*-, *m*-, and *o*-amidyl acetylenes and respective lysine conjugates.

In the present paper, we investigate the reactivity of three isomeric aryl-TFP alkynes with the amide substituent in different positions (*o*-, *m*-, and *p*-) relative to the alkyne (acetamides in Figure 4). Such variations in the substitution pattern are known to impose significant effects on photochemical reactivity [56–57]. Reactions with cyclohexadiene were used to probe the properties of the triplet excited states in these three isomers, whilst Stern–Volmer quenching experiments were used to investigate the kinetics of PET in these three systems. In the final part of this paper, we examine whether the observed trends in photochemical and photophysical properties correlate with DNA-cleaving activities of the corresponding lysine conjugates shown in Figure 4.

## Results and Discussion

### Synthesis

**Scheme 2:**
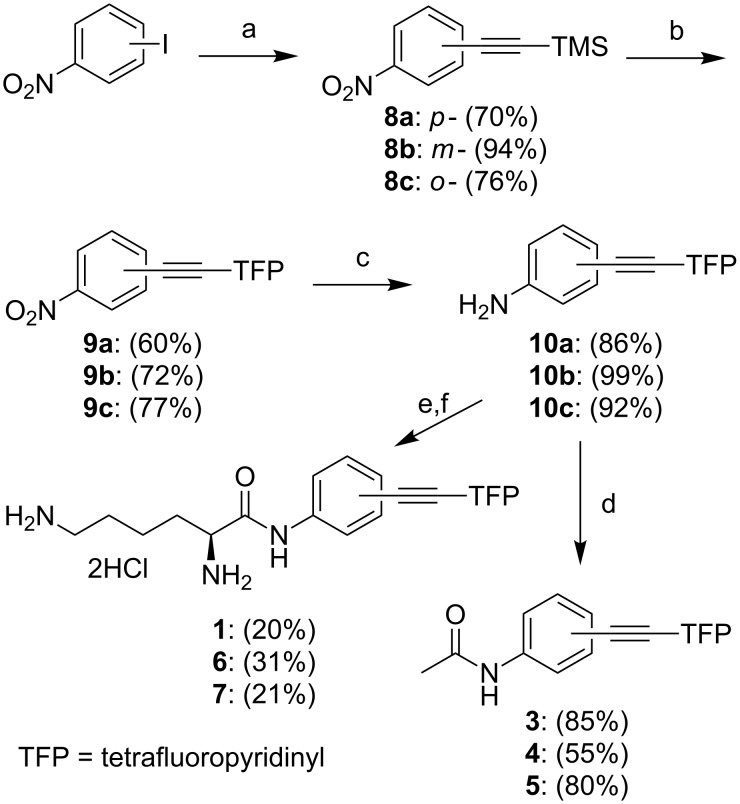
Synthesis of amido-substituted monoacetylenes and lysine conjugates. Reagents and conditions: a. PdCl_2_(PPh_3_)_2_, CuI, HCCSiMe_3_/Et_3_N, rt; b. CsF, pentfluoropyridine/DMF; c. SnCl_2_, EtOH, reflux; d. (CH_3_CO)_2_O, Et_3_N/CH_2_Cl_2_; e. POCl_3_, Boc-Lys(Boc)-OH/pyridine; f. HCl(g)/MeOH.

The regioisomeric diaryl alkynes were synthesized following the synthetic strategy previously outlined by us for compound **1** [25]. The Sonogashira coupling of the corresponding iodonitrobenzene with trimethylsilyl (TMS) acetylene produced acetylenes **8a**–**c**. The TMS group of acetylene **8** was directly substituted with a tetrafluoropyridyl (TFP) group by a CsF-promoted reaction with pentafluoropyridine in DMF. Reduction of the nitrobenzenes **9a**–**c** with SnCl_2_ produced anilines **10a**–**c**, which were reacted with acetyl chloride to form amides **3**, **4**, and **5** (Scheme 2).

Conjugates **1**, **6**, and **7** were prepared via coupling of the corresponding anilines **10a**–**c** with Boc-protected lysine in the presence of POCl_3_ in pyridine. The Boc groups were removed by treatment with gaseous HCl in MeOH.

### Photochemical reactions of TFP-alkynes with 1,4-cyclohexadiene

**Scheme 3:**
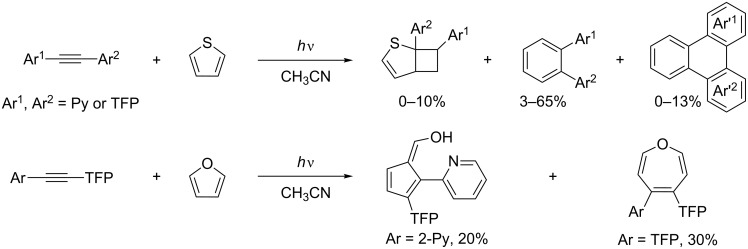
Photochemical reactions of TFP-substituted aryl alkynes with selected π-systems. In short, the reaction proceeds through the photoinduced electron transfer from thiophene to the singlet excited state of the diaryl acetylene. The initially formed cyclobutene product undergoes further photorearrangement via a formal 1,3-shift.

Previously, Zeidan and Alabugin have shown that TFP-substituted aryl alkynes are powerful photochemical alkylating agents and attack a variety of π-systems (Scheme 3) [58].

**Scheme 4:**
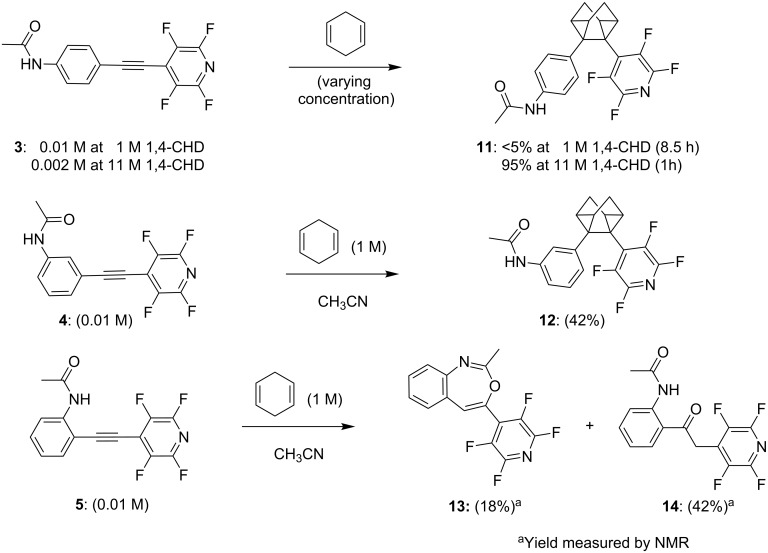
Photocycloaddition of amido acetylenes with 1,4-CHD.

We chose 1,4-CHD to probe alkyne photoreactivity because, similar to excited alkynes, 1,4-CHD displays multichannel reactivity and can act as a source of H-atoms, as a source of electrons in PET, or as a reactive π-system. Photocycloaddition of the three acetylene molecules with 1,4-CHD was investigated via irradiation in acetonitrile with a Luzchem LED photoreactor and UVB (310 nm) irradiation (Scheme 4). The *m*-substituted acetylene **4** provided the homoquadricyclane product **12** in 42% yield after 2 h of UV irradiation in the presence of 100 equiv of 1,4-CHD. Under the same conditions, the *p*-substituted acetylene **3** reacts with 1,4-CHD sluggishly and gave <5% of product after 8.5 h of UV irradiation according to the ^1^H NMR spectrum of the reaction mixture. This observation suggests that the ISC to the triplet state with *m*-acetamidyl acetylene **4** is more efficient than with *p*-acetamidyl acetylene **3**, the lifetime of the triplet of **4** is longer than that of **3**, or the triple state of **4** is more electrophilic than the triplet state of **3**. However, when the reaction was repeated in neat 1,4-CHD, the corresponding homoquadricyclane product **11** was isolated in 95% yield after only 1 h of UV irradiation. This result indicates that the photoaddition reaction of **3** can occur efficiently under more favorable conditions when there is a higher probability of intercepting the reactive excited state via reaction with a π-system.

**Scheme 5:**
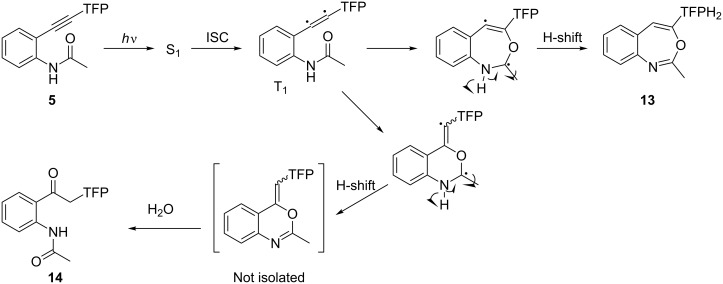
Possible mechanism for photochemical hydration of diaryl acetylene moiety catalyzed by the *ortho*-amide substituent.

The photochemical reactivity for the *o*-substituted acetylene **5** was drastically changed (Scheme 5). In this case, photoexcitation leads to the formation of an oxygen–carbon bond between the amide group and the triple bond. The cyclized product, benzoxazepine **13**, and the ketone product **14** were isolated. Whereas **13** was produced by a 7-endo cyclization (unprecedented in these systems), the ketone **14** can be formed either by direct hydration of the alkyne or by a known pathway that involves the corresponding six-membered product, a benzoxazine. The formation of benzoxazines has been previously reported by Roberts and coworkers, who suggested cyclization via triplet excitation following hydration [59–62]. The presence of vinyl peaks at 6.2 and 5.9 ppm in the reaction mixture and their quick disappearance upon the addition of a drop of water suggest that benzoxazines are also the intermediate products in our case but are rapidly hydrolyzed during work-up and purification. Although one can suggest the intermediacy of the triplet diradical in the photocyclization of *o*-amido acetylene **6**, this transformation does not require H-atom abstraction from an external H-atom source such as CHD and DNA, and thus the DNA-damaging ability of this chromophore is not expected to be significant.

### Photophysics and kinetics of photoinduced electron transfer

**Figure 5:**
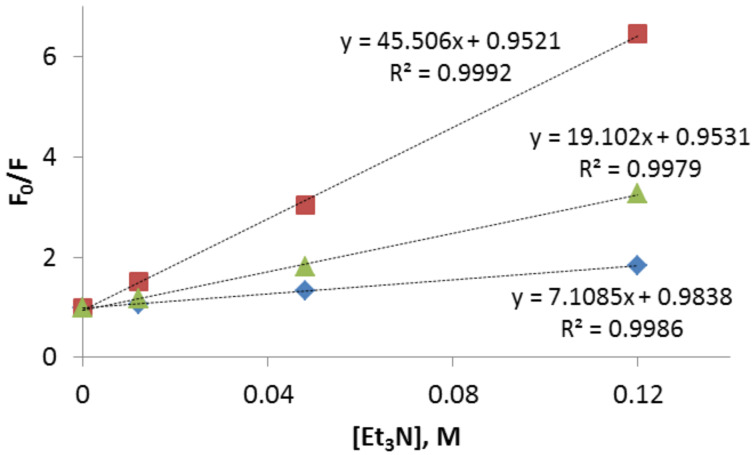
Stern–Volmer plots of three regioisomers, **3** (blue diamond), **4** (red square), and **5** (green triangle), in acetonitrile (10 μM). The solutions were excited at 310 nm.

The fluorescence quenching by triethylamine (Et_3_N) was examined in order to gauge the relative efficiencies of these compounds as DNA photo-oxidizers (Figure 5).

In the quenching experiments, the *meta*-isomer **4** showed the largest Stern–Volmer constant (*K*_sv_ = 45.51) among the three isomers, whereas the *para*-isomer **3** displayed the lowest efficiency of quenching. The measured singlet lifetimes allowed us to determine the quenching rate constant, *k*_q_, which, in this system, should be very close in magnitude to the rate of electron transfer, *k*_ET_ (Table 1).

**Table 1:** Stern–Volmer quenching constants (Et_3_N as a quencher) and singlet lifetimes for the isomeric acetylenes 3–5.

Compound	*K*_sv_ (M^−1^)	*τ* (ns)	*k*_q_ (M s^−1^)

**3** (*para*)	7.11	1.26 ± 3.22 × 10^−3^	5.64 × 10^9^
**4** (*meta*)	45.5	3.35 ± 9.30 × 10^−3^	1.36 × 10^10^
**5** (*ortho*)	19.1	1.34 ± 3.52 × 10^−3^	1.43 × 10^10^

The two- to three-fold increase in the rate of electron transfer from Et_3_N to the excited singlet state of the *meta*- and *ortho*-isomers in comparison to the *para*-isomer is consistent with the well-known photochemical *ortho*, *meta* effect of an acceptor substituent [56–57].

Although the fluorescence of all three isomers is quenched by the amine, the efficient quenching of singlet excitation in compound **4** can potentially lead to a stronger pH-dependency on the photochemistry of the respective lysine conjugate, which is controlled by the protonation-gated intramolecular electron transfer from the α-amino group [25]. Interestingly, the *meta*-isomer has a noticeably longer singlet lifetime than the other two isomers. A similar trend has been previously observed for the lifetimes of *m*-substituted enediynes [63].

**Figure 6:**
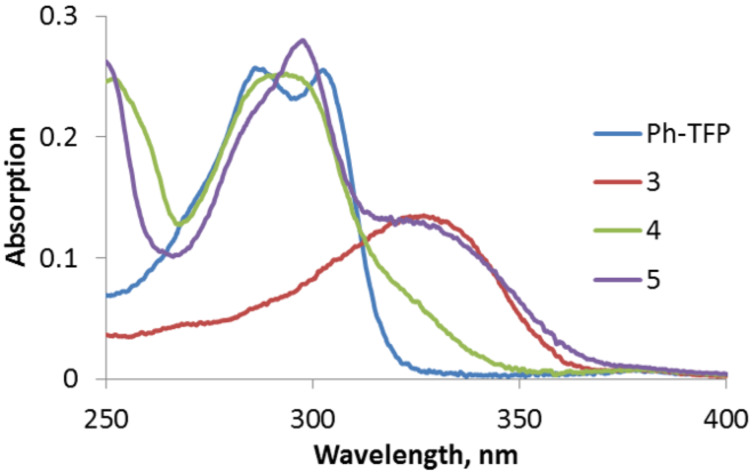
Absorption spectra of three isomers, **3**, **4**, **5**, and **Ph-TFP** in acetonitrile (10 μM).

The absorption spectra of all four acetylenes are shown in Figure 6. The core Ph-TFP-acetylene (**Ph-TFP**) chromophore without the amide group has no significant absorption at >320 nm.

The lowest absorptions of the *para*- and *ortho*-isomers **3**, **5** are red-shifted (λ_max_ ~ 330 nm) as a consequence of increased conjugation in the ground state. In contrast, the absorption of the *meta*-isomer **4** is closer to that of **Ph-TFP**, with the lower energy absorption band appearing as a lower-intensity shoulder.

### Efficiency of DNA photocleavage

**Figure 7:**
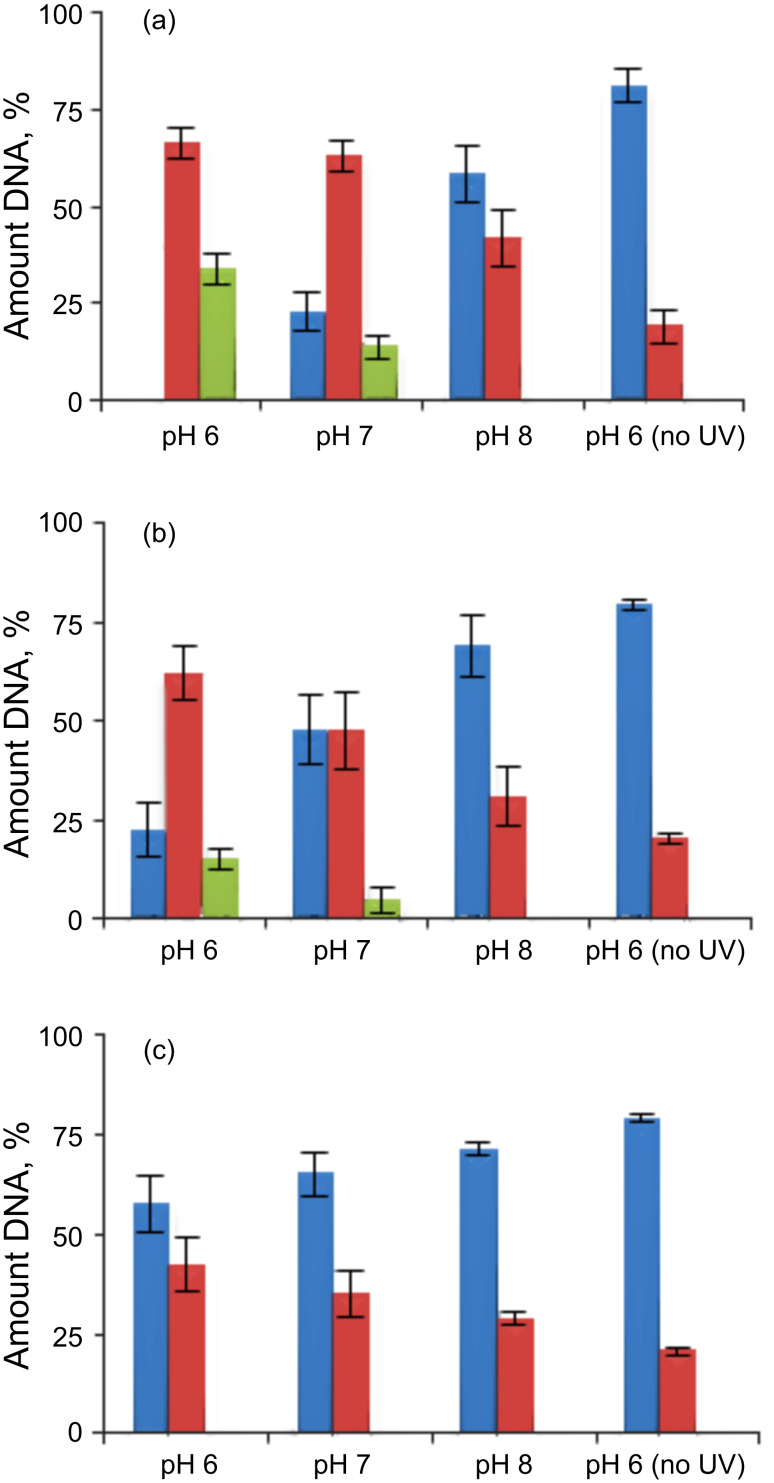
Quantified DNA cleavage data for **1** (a), **6** (b) and **7** (c). Blue: Form I (supercoiled) DNA; red: Form II (relaxed) DNA; green: Form III (linear) DNA. Reported values represent the average of four experiments.

The results of plasmid relaxation assay with three lysine conjugates are summarized in Figure 7.

These experiments were carried out on 15 μM of lysine conjugate with 30 μM/base pair of pBR322 plasmid DNA at pH 6, 7 and 8. The DNA-cleaving ability of conjugates does not directly follow the order of the photocycloaddition of their acetamides. Although the *m*-substituted acetylene was more photoreactive toward 1,4-CHD, the corresponding conjugate **6** produced less DNA cleavage than conjugate **1**. This suggests that either the difference in DNA binding overshadows the intrinsic differences in reactivity or the acetamide group is not a good surrogate for the lysine amides [64].

Nevertheless, both *p*- and *m*-lysine conjugates exhibit efficient ds DNA damage at pH 6 where the α-amino group of the lysine moiety is protonated and incapable of direct interference with the singlet photochemical process. On the other hand, compound **7**, which is unlikely to be a strong alkylating agent in the excited state, was the least-efficient DNA cleaver and did not produce any ds breaks. Interestingly, all three C-lysine conjugates broke DNA more efficiently at lower pH.

### Effects of radical scavengers on DNA cleavage

**Figure 8:**
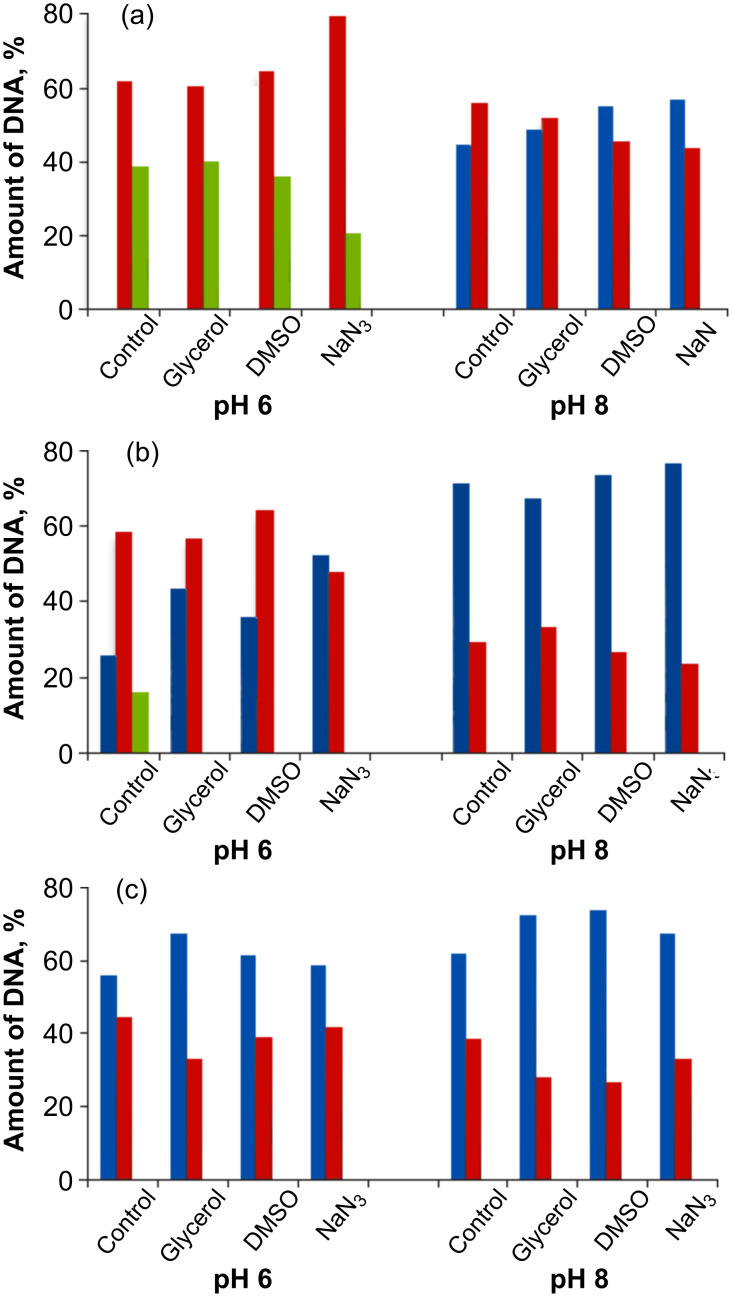
Effect of hydroxyl radical/singlet oxygen scavengers (20 mM) on the efficiency of DNA cleavage at pH 6 and 8 by 15 μM of conjugates **1** (a), **6** (b), and **7** (c) after 10 min of irradiation. Color coding: Blue: Form I (supercoiled) DNA; red: Form II (relaxed) DNA; green: Form III (linear) DNA.

In order to get further insight into the mechanism of the DNA cleavage by the three conjugates, we used the plasmid relaxation assays for the cleavage with conjugates **1**, **6**, and **7** in the presence of hydroxyl radicals (glycerol, DMSO) and singlet oxygen (NaN_3_) scavengers [65]. The results are summarized in Figure 8.

For compound **1** (Figure 8a), the hydroxyl radical scavengers have no effect at pH 6 while the singlet oxygen scavenger slightly decreases the amount of ds DNA cleavage. At pH 8, >10% of the protecting effect was observed for all of the scavengers. The protecting effect of the scavengers on the reactivity of conjugate **1** is insignificant considering the very large excess (>1000-fold) of the scavengers. Conjugate **1** still leaves no undamaged DNA and produces significant amounts of linear DNA at pH 6. This observation suggests that the main DNA damage mechanism by conjugate **1** is not sensitive to the presence of hydroxyl radical/singlet oxygen scavengers, which can only block the alternative minor mechanisms.

In contrast, the photocleavage by the *meta*-substituted conjugate **6** (Figure 8b) is inhibited by both types of scavengers among the three conjugates at pH 6. The hydroxyl radical scavengers, glycerol and DMSO, protected DNA from the cleavage by 33 and 26%, respectively, whereas NaN_3_ showed ~43% protection. The large protecting effect of NaN_3_, the singlet oxygen scavenger, is consistent with the efficient photoaddition reaction of its chromophore via triplet excitation. This suggests that *m*-conjugate is not tightly bound to DNA and the most damage is propagated via two different oxygen-centered species, likely to be generated via the triplet manifold. The hydroxyl radical scavengers protected DNA from ss DNA cleavage by compound **7**, but the effect was small (Figure 8c). Only glycerol at pH 6 and glycerol and DMSO at pH 8 showed ~10% of protection. Little effect was observed for NaN_3_, suggesting that the formation of singlet oxygen via triplet energy transfer is inefficient, possibly because of a short triplet lifetime and fast intramolecular photocyclization. The observed scavenger effects suggest different DNA damage mechanisms for the three lysine conjugates: Guanine oxidation and/or base alkylation for conjugate **1**, guanine oxidation and generation of reactive oxygen species for conjugate **6**, and guanine oxidation for conjugate **7**.

### Cell proliferation assay

**Figure 9:**
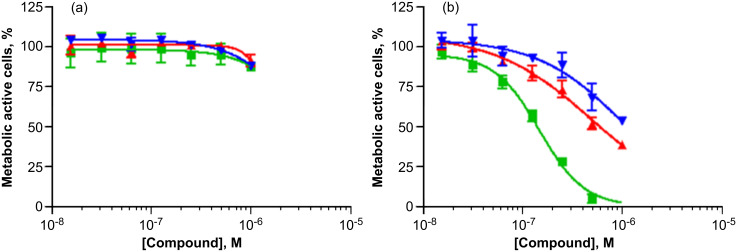
Cell proliferation assay using A375 cells (human melanoma) and compound **1** (green square), **6** (red up-pointing triangle), and **7** (blue down-pointing triangle) in dark (a) and after 10 min of UV (360 nm) irradiation.

The ability of compounds **1**, **6**, and **7** to inhibit cell proliferation in human melanoma cell lines was tested in the dark and under photoactivation (Figure 9).

According to the control experiments with all three conjugates in the dark, these compounds do not inhibit cell proliferation at concentrations of <1 μM. On the other hand, conjugate **1** displayed strong phototoxicity toward the human melanoma A375 cell line in the nanomolar range (CC_50_ = 1.49 × 10^−7^ M) after 10 min of UV irradiation at 360 nm. Conjugates **6** and **7** also showed some phototoxicity. This result of cell proliferation inhibition by the conjugates is consistent with their respective DNA-cleaving abilities.

## Conclusion

Three isomeric aryl-TFP alkynes with amide substituents in different positions (*o*-, *m*-, and *p*-) were synthesized, and the variations of their photochemical reactivity toward cyclohexadiene were investigated. Only *p*- and *m*-isomers were capable of alkylating 1,4-CHD. In contrast, the *o*-isomer only underwent an intramolecular reaction. The three analogous isomeric lysine conjugates cleaved DNA with different efficiencies: 15 μM of the *p*-, *m*-, and *o*-conjugates **1**, **6**, and **7** produced 34, 15, and 0% of ds DNA cleavage, respectively. The large DNA-protecting effect on reactivity of the *meta*-conjugate **6**, imposed by hydroxyl radical/singlet oxygen scavengers, suggests triplet photoreactivity which leads to efficient sensitization of singlet oxygen. This observation is consistent with the efficient triplet reactivity of its chromophore. The inhibition of human melanoma cell growth by the three conjugates was also tested. The *para*-substituted conjugate **1** has the lowest CC_50_ value of 1.49 × 10^−7^ M.

## Supporting Information

Supporting information features details for experimental procedures, emission titration spectra, fluorescence decay traces, picture of plasmid relaxation assay, characterization data, and NMR spectra (^1^H, ^13^C NMR, HSQC, and HMBC).

File 1 Experimental details, characterization data, emission titration spectra, fluorescence decay traces, plasmid relaxation assays and NMR spectra (^1^H, ^13^C NMR, HSQC, and HMBC).

## References

[1] Armitage B (1998). Chem Rev.

[2] Shiraki T, Sugiura Y (1990). Biochemistry.

[3] Jones G B, Wright J M, Plourde G, Purohit A D, Wyatt J K, Hynd G, Fouad F (2000). J Am Chem Soc.

[4] Kar M, Basak A (2007). Chem Rev.

[5] Kagan J, Wang X, Chen X, Lau K Y, Batac I V, Tuveson R W, Hudson J B (1993). J Photochem Photobiol, B: Biol.

[6] Benites P J, Holmberg R C, Rawat D S, Kraft B J, Klein L J, Peters D G, Thorp H H, Zaleski J M (2003). J Am Chem Soc.

[7] Schmittel M, Viola G, Dall’Acqua F, Morbach G (2003). Chem Commun.

[8] Poloukhtine A, Popik V V (2003). J Org Chem.

[9] Polukhtine A, Karpov G, Popik V V (2008). Curr Top Med Chem.

[R10] 10Alabugin, I. V.; Yang, W.-Y.; Pal, R. Enediyne photochemistry. In *CRC Handbook of Organic Photochemistry and Photobiology*, 3rd ed.; Taylor & Francis: Boca Raton, FL, in press.

[11] Celli J P, Spring B Q, Rizvi I, Evans C L, Samkoe K S, Verma S, Pogue B W, Hasan T (2010). Chem Rev.

[12] Alabugin I V, Timokhin V I, Abrams J N, Manoharan M, Abrams R, Ghiviriga I (2008). J Am Chem Soc.

[13] Pal R, Clark R J, Manoharan M, Alabugin I V (2010). J Org Chem.

[14] Zeidan T A, Kovalenko S V, Manoharan M, Alabugin I V (2006). J Org Chem.

[15] Pickard F C, Shepherd R L, Gillis A E, Dunn M E, Feldgus F, Kirschner K N, Shields G C, Manoharan M, Alabugin I V (2006). J Phys Chem A.

[16] Vasilevsky S F, Mikhailovskaya T F, Mamatyuk V I, Salnikov G E, Bogdanchikov G A, Manoharan M, Alabugin I V (2009). J Org Chem.

[17] Alabugin I V, Gilmore K, Patil S, Manoharan M, Kovalenko S V, Clark R J, Ghiviriga I (2008). J Am Chem Soc.

[18] Vasilevsky S F, Baranov D S, Mamatyuk V I, Gatilov Y V, Alabugin I V (2009). J Org Chem.

[19] Alabugin I V, Manoharan M (2005). J Am Chem Soc.

[20] Alabugin I V, Manoharan M (2005). J Am Chem Soc.

[21] Zeidan T, Manoharan M, Alabugin I V (2006). J Org Chem.

[22] Baroudi A, Mauldin J, Alabugin I V (2010). J Am Chem Soc.

[23] Alabugin I V, Manoharan M (2007). J Comput Chem.

[24] Kovalenko S V, Alabugin I V (2005). Chem Commun.

[25] Yang W-Y, Breiner B, Kovalenko S V, Ben C, Singh M, LeGrand S N, Sang Q-X, Strouse G F, Copland J A, Alabugin I V (2009). J Am Chem Soc.

[26] Breiner B, Schlatterer J C, Alabugin I V, Kovalenko S V, Greenbaum N L (2007). Proc Natl Acad Sci U S A.

[27] Yang W-Y, Cao Q, Callahan C, Galvis C, Sang Q-X, Alabugin I V (2010). J Nucleic Acids.

[28] Breiner B, Schlatterer J C, Kovalenko S V, Greenbaum N L, Alabugin I V (2006). Angew Chem, Int Ed.

[29] Nielsen P E, Jeepesen C, Egholm M, Buchardt O (1988). Nucleic Acids Res.

[30] Chatterjee M, Rokita S E (1990). J Am Chem Soc.

[31] Henriksen U, Larsen C, Karup G, Jeepesen C, Nielsen P E, Buchardt O (1991). Photochem Photobiol.

[32] Chatterjee M, Rokita S E (1994). J Am Chem Soc.

[33] Saito I, Takayama M, Sakurai T (1994). J Am Chem Soc.

[34] Nakatani K, Shirai J, Tamaki R, Saito I (1995). Tetrahedron Lett.

[35] Hosford M E, Muller J G, Burrows C J (2004). J Am Chem Soc.

[36] Turro N J, Evenzahav A, Nicolaou K C (1994). Tetrahedron Lett.

[37] Evenzahav A, Turro N J (1998). J Am Chem Soc.

[38] Kaneko T, Takanashi M, Hirama M (1999). Angew Chem, Int Ed.

[39] Funk R L, Young E R R, Williams R M, Flanagan M F, Cecil T L (1996). J Am Chem Soc.

[40] Choy N, Blanco B, Wen J, Krishan A, Russell K C (2000). Org Lett.

[41] Russell K C, Jones G B, Lenci F, Horspool W (2004). The Photo-Bergman Cycloaromatization of Enediynes. CRC Handbook of Organic Photochemistry and Photobiology.

[42] Spence J D, Hargrove A E, Crampton H L, Thomas D W (2007). Tetrahedron Lett.

[43] Zhao Z, Peacock J G, Gubler D A, Peterson M A (2005). Tetrahedron Lett.

[44] Wandel H, Wiest O (2002). J Org Chem.

[45] Nicolaou K C, Smith A L, Yue E W (1993). Proc Natl Acad Sci U S A.

[46] Galm U, Hager M H, Van Lanen S G, Ju J, Thorson J S, Shen B (2005). Chem Rev.

[47] Alabugin I V, Kovalenko S V (2002). J Am Chem Soc.

[48] Alabugin I V, Manoharan M (2003). J Am Chem Soc.

[49] Alabugin I V, Breiner B, Manoharan M (2007). Adv Phys Org Chem.

[50] Prall M, Wittkopp A, Schreiner P R (2001). J Phys Chem A.

[51] Vavilala C, Byrne N, Kraml C M, Ho D M, Pascal R A (2008). J Am Chem Soc.

[52] Ramkumar D, Kalpana M, Varghese B, Sankararaman S, Jagadeesh M N, Chandrasekhar J (1996). J Org Chem.

[53] Zeidan T A, Kovalenko S V, Manoharan M, Clark R J, Ghiviriga I, Alabugin I V (2005). J Am Chem Soc.

[54] Zeidan T A, Clark R J, Ghiviriga I, Kovalenko S V, Alabugin I V (2005). Chem–Eur J.

[55] Zhou Z, Fahrni C J (2004). J Am Chem Soc.

[56] Zimmerman H E (1995). J Am Chem Soc.

[57] Zimmerman H E, Alabugin I V (2001). J Am Chem Soc.

[58] Zeidan T A (2005). Thermal and Photochemical Reactions of Acetylenes: I-Ortho-Effect in the Bergman Cyclization of Benzannelated Enediynes II-Photocycloaddition of Diaryl Acetylenes to Cyclic Dienes Mechanisms and Applications.

[59] Roberts T D, Ardemagni L, Shechter H (1969). J Am Chem Soc.

[60] Munchausen L, Ookuni I, Roberts T D (1971). Tetrahedron Lett.

[61] Staudenmayer R, Roberts T D (1974). Tetrahedron Lett.

[62] Roberts T D, Munchausen L, Shechter H (1975). J Am Chem Soc.

[63] Kauffman J F, Turner J M, Alabugin I V, Breiner B, Kovalenko S V, Badaeva E A, Masunov A, Tretiak S (2006). J Phys Chem A.

[R64] 64We have shown before that the α-amino group (which is missing in the acetamides) has an effect on the reactivity. See Ref. [25].

[65] Devasagayam T P A, Steenken S, Obendorf M S W, Schulz W A, Sies H (1991). Biochemistry.

