# Supplementary material for: Fine-tuning alkyne cycloadditions: Insights into photochemistry responsible for the double-strand DNA cleavage via structural perturbations in diaryl alkyne conjugates
Source: Beilstein J Org Chem. 2011 Jun 16;7:813–23. doi: 10.3762/bjoc.7.93 (PMC3135214; doi:10.3762/bjoc.7.93)
Supplement: File 1 — Experimental details, characterization data, emission titration spectra, fluorescence decay traces, plasmid relaxation assays and NMR spectra (1H, 13C NMR, HSQC, and HMBC). [file Beilstein_J_Org_Chem-07-813-s001.pdf]

Supporting Information

for

Fine-tuning alkyne cycloadditions: Insights into photochemistry responsible for the double-strand DNA cleavage via structural perturbations in diaryl alkyne conjugates

Wang-Yong Yang, Samantha A. Marrone, Nalisha Minors, Diego A. R. Zorio and Igor V. Alabugin*

Address: Department of Chemistry and Biochemistry, Florida State University, Tallahassee, FL 32306-4390, USA

Email: Wang-Yong Yang - yang@chem.fsu.edu; Igor V. Alabugin* - alabugin@chem.fsu.edu; Diego A. R. Zorio - zorio@chem.fsu.edu

* Corresponding author

Experimental details, characterization data, emission titration spectra, fluorescence decay traces, plasmid relaxation assays and NMR spectra (^{1}H, ^{13}C NMR, HSQC, and HMBC).

TABLE OF CONTENTS

Experimental Details.. S2

Characterization Data.. S4

Figure S1: Emission titration of **6** (a), **7** (b) and **8** (c) in acetonitrile (10 μM) with 0, 0.012, 0.048 and 0.12 M of Et_3N. Excitation: 330 **nm**. S11

Figure S2: The fluorescence decay traces of 3 (a), 4 (b) and 5 (c) in acetonitrile................ S12

Table S1: Analyzed fluorescence decay data for three isomers **3, 4** and **5**.......................... S13

Figure S3: Picture of plasmid relaxation assay with 15 µM of lysine conjugates, 1, 6 and 7, and pBR 322 plasmid DNA (30 µM/bp) in 20 mM sodium phosphate buffer after 10 min of irradiation. Form I: intact supercoiled DNA, form II: relaxed form (ss cleavage), form III: linear form (ds cleavage) S13

Figure S4: Quantitative plots of plasmid relaxation assays with 30 µM (b.p.) of pBR 322 after 10 min. of UV (>300 nm wavelength) irradiation at pH 6, 7 and 8 .... S13

NMR Spectra S14

References... S30

General information. ^{1}H, ^{13}C NMR spectra were recorded on a Varian Gemini 300 MHz, Bruker 400 MHz and 600 MHz NMR spectrometer. Mass spectrometry data was collected on a Jeol JMS-600H. UV spectra were recorded on a Shimadzu UV-2100. Fluorescence spectra were obtained with SPEX FluoMax spectrofluorimeter using right-angle geometry. pH was monitored with AB 15 plus pH meter (Accument) after standardization at 25 ºC. All buffers were prepared and pH was adjusted with HCl (aq.) and NaOH (aq.) at room temperature (25 °C).

Photochemical reactions of amidyl acetylenes with 1.4-cyclohexadiene. Solutions of the acetylene and 1,4-CHD in acetonitrile were degassed with argon gas and the samples were placed into the photoreactor (Luzchem, LZC-4X photoreactor with 14 LZC-UVB (310 nm, 8 W) lamps).

Singlet excitation lifetime. The singlet excitation lifetimes were measured using the time-correlated single photon counting (TCSPC) technique. The samples were excited at 295 nm wavelength with LED operating at a repetition rate of 1MHz. The emission decay was observed at emission λ_{max} of each sample and data were recorded with 10,000 counts in the peak channel. The timescale of the experiment was 200 ns (29.19 ps/channel). The decay data were analyzed with DAS6 software.

Plasmid DNA photocleavage. pBR322 plasmid DNA (4,361 b/p; from BioLabs Inc., 1µg/µL solution in 10 mM Tris-HCl (pH 8.0), and 1mM EDTA buffer) was diluted to a concentration of

0.01 μg/μL. The solution containing the cleavage agent, DNA (30 μM/bp) in 20 mM sodium phosphate buffer was incubated for 1 hour at 30 °C. Samples were placed in ice at a distance of 20 cm from 200 W Hg-Xe lamp (Spectra-Physics, Laser & Photonics Oriel Instruments with long pass filter with 300 nm cut-on wavelength).

Electrophoretic analysis. The gel electrophoresis was carried out in 1x TBE buffer at 80 V using Miligel FisherBiotech Horizontal Electrophoresis System. All gels were run on 1% agarose slab gels. Before loading, the DNA samples were mixed with 0.33 volume of tracking dye containing bromophenol blue (0.25%) and glycerol (30%) in water. After staining in ethidium bromide solution (2 μg/ml) for 1 hour, the gel was washed with water and pictures were taken. The relative quantities of the supercoiled, nicked, and linear DNA were calculated by integrating the "area" of each spot by the image analyzer software Total/Lab (Nonlinear Dynamics Ltd., UK). The amount of supercoiled DNA was multiplied by factor of 1.4 to account for reduced ethidium bromide intercalation into supercoiled DNA.

Cell culture. The human cancer cell line used in this report was obtained from the American Type Culture Collection (ATCC®). The human mesothelioma cell line MSTO-211H (CRL-2081™) was cultured in RPMI 1640 medium supplemented with 10% fetal bovine serum (FBS) and penicillin/streptomycin (Life Technologies™). Cells were propagated according to ATCC guidelines and maintained in a 37 °C incubator with 5% CO_2 atmosphere.

MTT assay. A375 cells were seeded at 2000 cells per well in a 96-well plate. Seven 2-fold serial dilutions of the compounds were added to the cells in triplicate. Concentrations were from 1 to .016 μM of compounds. After incubation for 4h with the compounds, cells were UV-irradiated for ten minutes at 365 nm (UV transluminator, Spectronomics Corp. model TR-365R). After 72hs of incubation, MTT (Sigma® cat# M2128) was added to cells to give a final concentration of 1.25 mg/ml and incubated for 60 minutes. Cells were centrifuged at 900 g for 5 minutes at room temperature. Media was replaced with DMSO and absorbance measured in a plate reader at 570 nm.

Synthesis of compounds. All reagents used were obtained from commercial sources and were of the highest grade available.

Trimethyl[(3-nitrophenyl)ethynyl]silane (**8b**)

A mixture of 3-iodonitrobenzene (2.0 g, 8.0 mmol), bis(triphenylphosphine)palladium(II) chloride (0.30 g, 0.40 mmol) and copper(I) iodide (0.080 g, 0.40 mmol) in 25 ml of Et_3N was degassed by freeze/pump/thaw technique (three times). Trimethylsilylacetylene (1.0 g, 10 mmol) was added and the mixture stirred for 12 hours. The reaction mixture was filtered through a celite pad and the pad rinsed with CH_2Cl_2. The filtrate was washed successively with sat. NH_4Cl(aq.) and brine. The organic layer was dried (Na_2SO_4), filtered, and concentrated. The residue was purified by column chromatography (hexane, then EtOAc:hexanes = 1:30) to afford trimethyl[(3-nitrophenyl)ethynyl]silane (1.65 g, 94%): ^{1}H NMR (300 MHz, $CDCl_3$) δ 8.32 (dd, *J* = 1.8, 1.8 Hz, 1H), 8.17 (ddd, *J* = 8.4, 2.1, 0.9 Hz, 1H), 7.76 (ddd, *J* = 7.5, 1.2, 1.2 Hz, 1H), 7.49 (dd, *J* = 8.1, 8.1 Hz, 1H), 0.28 (s, 9H).[1]

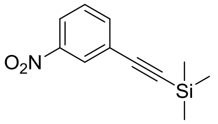

Trimethyl[(2-nitrophenyl)ethynyl]silane (**8c**)

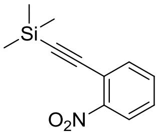

A mixture of 2-iodonitrobenzene (2.0 g, 8.0 mmol), bis(triphenylphosphine)palladium(II) chloride (0.30 g, 0.40 mmol) and copper(I) iodide (0.080 g, 0.40 mmol) in 25 ml of Et_3N was degassed by freeze/pump/thaw technique (three times). Trimethylsilylacetylene (1.0 g, 10 mmol) was added and the mixture stirred for 18 hours. The reaction mixture was filtered through a celite pad and the pad was rinsed with CH_2Cl_2. The filtrate was washed successively with sat. NH_4Cl(aq.) and brine. The organic layer was dried (Na_2SO_4), filtered, and concentrated. The residue was purified by column chromatography (hexane, then EtOAc:hexane = 1:60, 1:30) to afford trimethyl[(2-nitrophenyl)ethynyl]silane (1.33 g, 76%): ^{1}H NMR (300 MHz, $CDCl_3$) δ 8.02 (dd, *J* = 8.1, 1,2 Hz, 1H), 7.66 (dd, *J* = 7.5, 1.5 Hz, 1H), 7.56 (ddd, *J* = 7.2, 7.2, 1.2 Hz, 1H), 7.45 (ddd, *J* = 8.1, 8.1, 1.5 Hz, 1H), 0.28 (s, 9H).[2]

2,3,5,6-Tetrafluoro-4-8(3-nitrophenyl)ethynyl]pyridine (**9b**)

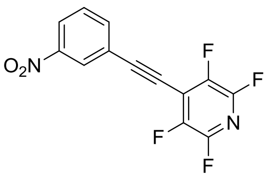

A solution of trimethyl[(3-nitrophenyl)ethynyl]silane, **8b** (1.45 g, 6.61 mmol) in DMF (10 mL) was added slowly to a mixture of pentafluoropyridine (1.45 g, 8.59 mmol) and CsF (1.51 g, 9.92 mmol) in DMF (10 mL). The reaction mixture was stirred overnight. Brine (30 mL) and dichloromethane (60 mL) were added. The organic phase was

separated and washed with water (30 ml × 3). The solvent was evaporated by rotary evaporation and the residue purified by column chromatography (hexane, then EtOAc:hexane = 1:30) to give the title compound in 72% yield: ^{1}H NMR (300 MHz, $CDCl_3$) δ 8.49 (dd, *J* = 1.8, 1.8 Hz, 1H), 8.35 (ddd, *J* = 8.4, 2.1, 0.9 Hz, 1H), 7.96 (ddd, *J* = 7.8, 1.2, 1.2 Hz, 1H), 7.66 (dd, *J* = 8.1, 8.1 Hz, 1 H); ^{13}C NMR (75 MHz, $CDCl_3$) δ 148.2, 143.5 (dm, *J* = 247.6 Hz), 141.9 (dm, *J* = 264.0 Hz), 129.9, 127.0, 125.1, 122.2, 116.3 (tt, *J* = 16.0, 4.4 Hz), 102.9 (t, *J* = 3.5 Hz), 75.2 (t, *J* = 4.2 Hz); HRMS (CI+): calcd for $C_{13}H_5F_4N_2O_2$ $[M + H]^+$ 297.02872, found 297.02908; m.p. 130-131 °C.

2,3,5,6-Tetrafluoro-4-[(2-nitrophenyl)ethynyl]pyridine (**9c**)

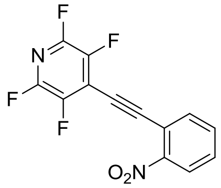

A solution of trimethyl[(2-nitrophenyl)ethynyl]silane, **8c** (1.10 g, 5.02 mmol) in DMF (10 mL) was added slowly to a mixture of pentafluoropyridine (1.08 g, 6.53 mmol) and CsF(1.10 g, 7.52 mmol) in DMF (10 mL). The reaction mixture was stirred overnight. Brine (30 mL) and dichloromethane (60 mL) were added. The organic phase was separated and washed with water (30 ml × 3). The solvent was evaporated by rotary evaporation and the residue purified by column chromatography (hexane, then EtOAc:hexane = 1:30) to give the title compound in 77% yield: ^{1}H NMR (300 MHz, $CDCl_3$) δ 8.24 (dd, *J* = 7.8, 1.5 Hz, 1H), 7.85 (dd, *J* = 7.5, 1.5 Hz, 1H), 7.73 (ddd, *J* = 7.5, 7.5, 1.5 Hz, 1H), 7.66 (ddd, *J* = 7.8, 7.8, 1.5 Hz, 1H); ^{13}C NMR (150 MHz, $CDCl_3$) δ 149.7, 143.7 (dm, *J* = 234.1 Hz), 142.1 (dm, *J* = 264.7 Hz), 135.5, 133.5, 131.2, 125.3, 116.7 (t, *J* = 16.3 Hz), 116.2, 100.9 (t, *J* = 3.2 Hz), 79.8 (t, *J* = 4.1 Hz); HRMS (CI+): calcd for $C_{13}H_5F_4N_2O_2$ $[M + H]^+$ 297.02872, found 297.02911; m.p. 109-110 °C.

3-[(Perfluoropyridin-4-yl)ethynyl]aniline (**10b**)

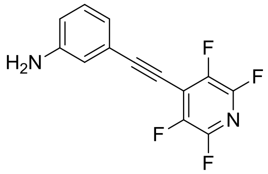

Stannous chloride (3.94 g, 20.8 mmol) was added to a solution of 2,3,5,6-tetrafluoro-4-[(3-nitrophenyl)ethynyl]pyridine, **9b** (1.23 g, 4.15 mmol) in EtOH (100 ml). The reaction mixture was heated under refluxed for 1.5 hours. The mixture was made basic (pH >9) with NaOH (1.0 N solution) and the product extracted with dichloromethane. The solvent was evaporated and 0.73 g (66%) of the product was obtained by recrystallization from benzene. The filtrate was concentrated and a further 0.37 g of the product was obtained by column chromatography on silica gel with EtOAc:hexane = 1:5 as eluent (overall yield 99%): ^{1}H NMR (300 MHz, $CDCl_3$) δ 7.21 (dd, *J* = 7.2, 7.2 Hz, 1H), 7.03 (d, *J* = 7.5 Hz, 1H), 6.93 (s, 1H), 6.79 (dd, *J* = 7.4, 1.5 Hz, 1H), 3.80 (s, 2H); ^{13}C NMR (150 MHz, $CDCl_3$) δ 146.7, 143.6 (dm, *J* = 241.8 Hz), 142.0 (dm, *J* = 262.4 Hz), 129.8, 122.8, 121.4, 118.2, 117.7 (m), 117.6, 107.4 (t, *J* = 3.8 Hz), 72.9 (t, *J* = 4.3 Hz); HRMS (ESI+): calcd for $C_{13}H_7F_4N_2$ $[M + H]^+$ 267.05454, found 267.05405; m.p. 172-173 °C.

2-[(Perfluoropyridin-4-yl)ethynyl]aniline (10c)

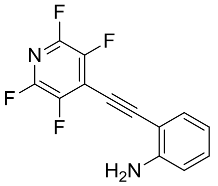

Stannous chloride (3.26 g, 17.2 mmol) was added to a solution of 2,3,5,6-tetrafluoro-4-[(2-nitrophenyl)ethynyl]pyridine, **9c** (1.02 g, 3.44 mmol) in EtOH (100 ml). The reaction mixture was heated under refluxed for 1.5 hours. The mixture was made basic (pH >9) with NaOH (1.0 N solution) and the product extracted with dichloromethane. The solvent was evaporated and 0.22 g (24%) of the product aniline was obtained by recrystallization from benzene. The filtrate was concentrated and a further (0.53 g, 68%) of the product was obtained by column chromatography on silica gel using EtOAc:hexane = 1:10 as eluent: ^{1}H NMR (300 MHz, acetone-d$_6$) δ 7.39 (d, *J* = 7.5 Hz, 1H), 7.26 (ddd, *J* = 8.4, 6.9, 1.5 Hz, 1H), 6.89 (d, *J* = 8.4 Hz, 1H), 6.69 (ddd, *J* = 7.5, 7.5, 1.2 Hz, 1H), 5.35 (s, 2H); ^{13}C NMR (150 MHz, $CDCl_3$) δ 149.3, 143.7 (dm, *J* = 245.3 Hz), 141.4 (dm, *J* = 260.9 Hz), 133.0, 132.6, 118.3, 117.8 (m), 114.8, 104.7, 104.6 (t, *J* = 3.5 Hz), 79.4 (t, *J* = 4.3 Hz); HRMS (ESI+): calcd for $C_{13}H_7F_4N_2$ $[M + H]^+$ 267.05454, found 267.05454; m.p. 133-134 °C.

***N*-{4-[(Perfluoropyridin-4-yl)ethynyl]phenyl}acetamide (3)**

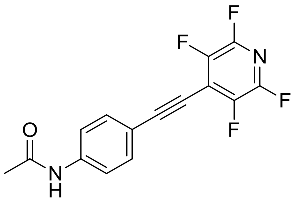

To a solution of 4-[(perfluoropyridin-4-yl)ethynyl]aniline **10a** (0.158 g, 0.59 mmol) in 3 ml of CH_2Cl_2, were added acetic anhydride (0.134 g, 1.26 mmol) and Et_3N (0.128 g, 1.26 mmol), and the reaction mixture was stirred at room temperature overnight. After concentrating the solution in vacuo, the product was isolated by column chromatography (EtOAc:hexane = 1:2 to 1:1) in 85% yield: ^{1}H NMR (400 MHz, CD_3OD) δ 7.70 (d, *J* = 8.6 Hz, 2H), 7.59 (d, *J* = 8.6 Hz, 2H), 2.15 (s, 3H); ^{13}C NMR (150 MHz, CD_3CN) δ 170.1, 144.5 (dm, *J* = 237 Hz), 143.1 (dm, *J* = 259 Hz), 142.7, 134.2, 120.0, 115.4, 107.5 (t, *J* = 3.4 Hz), 73.6 (t, *J* = 4.3 Hz), 24.5; HRMS (ESI+): calcd for $C_{15}H_9F_4N_2O$ $[M + H]^+$ 309.06510, found 309.06678; m.p. 189-190 °C.

***N*-{3-[(Perfluoropyridin-4-yl)ethynyl]phenyl}acetamide (4)**

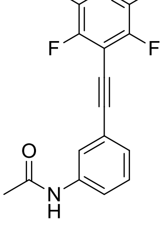

To a solution of 3-[(perfluoropyridin-4-yl)ethynyl]aniline **10b** (0.200 g, 0.751 mmol) in 3 ml of CH_2Cl_2, were added acetic anhydride (0.116 g, 1.13 mmol) and Et_3N (0.114 g, 1.13 mmol) and the reaction mixture was stirred at room temperature overnight. After concentrating the solution in vacuo, the product was isolated by column chromatography (EtOAc:hexane = 1:2 to 1:1) in 55% yield: ^{1}H NMR (400 MHz, CD_3OD) δ 7.96 (s, 1H), 7.64 (d, *J* = 8.0 Hz, 1H), 7.42 (dd, *J* = 7.7 Hz, *J* = 7.7 Hz, 1H), 7.36 (d, *J* = 7.7 Hz, 1H), 2.15 (s, 3H); ^{13}C NMR (100 MHz, $CDCl_3$) δ 168.5, 143.6 (dm, *J* = 249 Hz), 142.0 (dm, *J* = 263 Hz), 138.4, 129.6, 128.3, 123.3, 122.1, 121.4,

117.3 (m), 106.2 (m), 73.6 (m), 24.8; HRMS (ESI+): calcd for $C_{15}H_9F_4N_2O$ [M + H]$^+$ 309.06510, found 309.06584; m.p. 215-216 °C.

N-{2-[(Perfluoropyridin-4-yl)ethynyl]phenyl}acetamide (5)

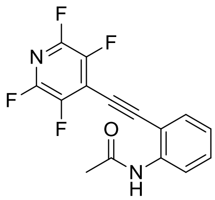

To a solution of 3-[(perfluoropyridin-4-yl)ethynyl]aniline **10b** (0.078 g, 0.29 mmol) in 1.5 ml of CH_2Cl_2, were added acetic anhydride (0.044 g, 0.44 mmol) and Et_3N (0.044 g, 0.44 mmol) and the reaction mixture was stirred at room temperature for 1day. After concentrating the solution in vacuo, the product was isolated by column chromatography (EtOAc:hexane = 1:3 to 1:1) in 80% yield: ^{1}H NMR (400 MHz, CD_3CN) δ 8.24 (brs, 1H), 8.09 (d, *J* = 7.9 Hz, 1H), 7.64 (d, *J* = 7.9 Hz, 1H), 7.53 (dd, *J* = 7.3 Hz, *J* = 7.3 Hz, 1H), 7.22 (dd, *J* = 7.6 Hz, *J* = 7.6 Hz, 1 H), 2.16 (s, 3H); ^{13}C NMR (100 MHz, $CDCl_3$) δ 168.5, 143.9 (dm, *J* = 199.1 Hz), 141.4 (dm, *J* = 220.5 Hz), 140.3, 132.7, 132.5, 123.9, 120.1, 116.9 (m), 109.2, 102.3, 80.7, 24.9; HRMS (ESI+): calcd for $C_{15}H_9F_4N_2O$ [M + H]$^+$ 309.06510, found 309.06417; m.p. 204.2-205 °C.

(*S*)-2,6-Diamino-*N*-{3-[(perfluoropyridin-4-yl)ethynyl]phenyl}hexanamide dihydrochloride (6)

L-Boc-Lys(Boc)-OH (0.26 g, 0.75 mmol) was dissolved in 3 ml of pyridine. The solution was cooled to –25 °C and phosphorus oxychloride (0.12 g, 0.75 mmol) added dropwise with vigorous

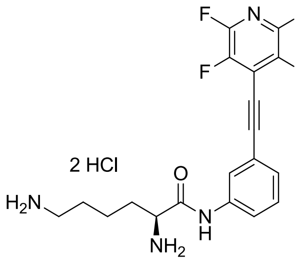

stirring. After stirring for 15 min at –25 °C, 3-[(perfluoropyridin-4-yl)ethynyl]aniline **10b** (0.10 g, 0.36 mmol) in pyridine (3 ml) was added slowly. The reaction mixture was stirred for 0.5 h at –25 °C and then at room temperature for 10 h. The reaction was quenched with ice/water and extracted with EtOAc. The organic layer was washed with sat. $NaHSO_4$ three times, dried with Na_2SO_4 and concentrated in vacuo. The crude product was purified by chromatography with CH_2Cl_2:CH_3CN = 1:30 to give the desired product in 36% yield. The latter (97 mg, 0.16 mmol) was dissolved in MeOH saturated with gaseous HCl (4 ml) at 0 °C and the solution stirred for 10 h at room temperature. The solvent was evaporated and the product purified by recrystallization from isopropanol to give the title compound (65 mg, 86%) was: ^{1}H NMR (600 MHz, CD_3OD) δ 8.07 (dd, *J* = 1.74 Hz, *J* = 1.68 Hz, 1H), 7.75 (ddd, *J* = 7.98 Hz, *J* = 1.92 Hz, *J* = 1.14 Hz, 1H), 7.48 (dd, *J* = 7.8 Hz, *J* = 7.8 Hz, 1H), 7.43 (ddd, *J* = 7.68 Hz, *J* = 1.32 Hz, *J* = 1.32 Hz, 1H) 4.12 (t, *J* = 6.6 Hz, 1H), 2.97 (t, *J* = 7.56 Hz, 2H), 2.00-2.07 (m, 1H), 1.97-2.00 (m, 1H), 1.74-1.77 (m, 2H), 1.56-1.60 (m, 2H); ^{13}C NMR (150 MHz, CD_3OD) δ 168.7, 144.9 (dm, *J* = 224 Hz), 143.5 (dm, *J* = 262 Hz), 139.7, 130.8, 129.4, 124.2, 123.3, 122.4, 117.9 (m), 106.5 (m), 74.2 (m), 54.9, 40.3, 32.2, 28.2, 23.0; HRMS (ESI+): calcd for $C_{19}H_{19}F_4N_4O$ [M + H]$^+$ 395.14950, found 395.14977; m.p. >250 °C (decomp.)

(*S*)-2,6-Diamino-*N*-(2-((perfluoropyridin-4-yl)ethynyl)phenyl)hexanamide dihydrochloride (7)

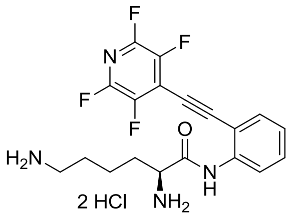

L-Boc-Lys(Boc)-OH (0.52 mg, 1.5 mmol) was dissolved in 3 ml of pyridine. The solution was cooled to –25 °C and phosphorus oxychloride (0.23 g, 1.5 mmol) was added dropwise with vigorous stirring. After stirring for 15 min. at –25 °C, 2-[(perfluoropyridin-4-yl)ethynyl]aniline **10c** (0.20 g, 0.75 mmol) in pyridine (3 ml) was added slowly. The reaction mixture was stirred for 0.5 h at –25 °C and then at room temperature for 10 h. The reaction was quenched with ice/water and extracted with EtOAc. The organic layer was washed with sat. $NaHSO_4$ three times, dried with Na_2SO_4 and concentrated in vacuo. The crude product was purified by chromatography with CH_2Cl_2:CH_3CN = 1:30 to give the desired product in 24% yield. The latter (185 mg, 0.31 mmol) was dissolved in MeOH saturated with gaseous HCl (6 ml) at 0 °C and the solution stirred for 10 h at room temperature. The solvent was evaporated and the product was purified by recrystallization from isopropanol to give the title compound (124 mg, 86%): ^{1}H NMR (300 MHz, CD_3OD) δ 7.89 (d, *J* = 8.1 Hz, 1H), 7.72 (dd, *J* = 7.8, 1.5 Hz, 1H), 7.58 (ddd, *J* = 7.2, 1.2, 1.5 Hz, 1H), 7.36 (ddd, *J* = 7.2, 7.2, 1.2 Hz, 1H), 4.20 (t, *J* = 6.2 Hz, 1H), 2.96 (t, *J* = 7.5 Hz, 2H), 1.98-2.19 (m, 2H), 1.71-1.81 (m, 2H), 1.56-1.70 (m, 2H); ^{13}C NMR (100 MHz, CD_3OD) δ 169.1, 144.9 (dm, *J* = 240.9 Hz), 143.2 (dm, *J* = 259.8 Hz), 139.5, 134.6, 132.8, 127.4, 125.9, 118.1 (m), 116.0, 103.2 (t, *J* = 3.6 Hz), 79.6 (t, *J* = 4.3 Hz), 54.8, 40.3, 32.2, 28.2, 23.1; HRMS (CI+): calcd for $C_{19}H_{19}F_4N_4O$ $[M + H]^+$ 395.1495, found 395.1487; m.p. 222-223 °C (decomp.)

***N*-(4-(2a^1-(perfluoropyridin-4-yl)octahydrodicyclopropa[*cd,gh*]pentalen-1a^1-yl)phenyl)acetamide (11)**

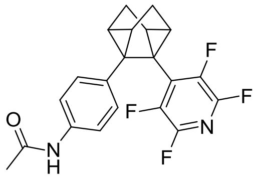

A solution of *N*-{4-[(perfluoropyridin-4-yl)ethynyl]phenyl}acetamide (**3**) (5 mg, 0.016 mmol) in 8 ml of 1,4-cyclohexadiene was degassed by bubbling Ar into the solution for 20 minutes. After 1 hour UV (310 nm) irradiation, the solution was concentrated in vacuo and the product was purified by column chromatograpy (EtOAc:hexane = 1:2) to give the title compound in 95% yield: ^{1}H NMR (400 MHz, $CDCl_3$) δ 7.29 (d, *J* = 8.5 Hz, 2H), 7.17 (d, *J* = 8.4 Hz, 2H), 7.01 (s, 1H), 2.32 (dm, *J* = 13.0 Hz, 2H), 1.9-2.0 (m, 4H), 1.84 (d, *J* = 1.7 Hz, 2H); ^{13}C NMR (150 MHz, CD_3OD) δ 168.15, 143.17 (dm, *J* = 236.6 Hz), 141.90 (dm, *J* = 254.6 Hz), 136.74, 134.30, 134.19, 130.57, 119.63, 43.39, 33.99, 33.07, 32.93, 29.83, 24.71, 24.40; HRMS (ESI+): calcd for $C_{21}H_{16}F_4N_2NaO$ $[M + Na]^+$ 411.10965, found 411.10981.

***N*-(3-(2a[1]-(perfluoropyridin-4-yl)octahydrodicyclopropa[*cd,gh*]pentalen-1a[1]-yl)phenyl)acetamide** (**12**)

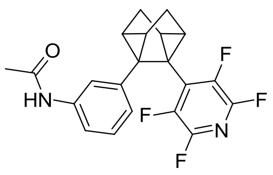

N-{3-[(Perfluoropyridin-4-yl)ethynyl]phenyl}acetamide (**4**) (5 mg, 0.016 mmol) and 1,4-cyclohexadiene (130 mg, 1.6 mmol) were dissolved in 1.6 ml of acetonitrile. After degassing by bubbling Ar into the solution for 10 minutes, the reaction mixture was irradiated under UV (310 nm) for 2 hours. The solvent was removed in vacuo and the desired product (42%) was obtained by column chromatograpy (EtOAc:hexane = 1:2): ^{1}H NMR (400 MHz, $CDCl_3$) δ 7.35 (s, 1H), 7.23 (d, *J* = 8.6 Hz, 1H), 7.10 (dd, *J* = 7.8 Hz, *J* = 7.8 Hz, 1H), 6.95 (d, *J* = 7.4 Hz, 1H), 2.32 (dm, *J* = 13.0 Hz, 2H), 1.94-2.0 (m, 4H), 1.88 (d, *J* = 1.7 Hz, 2H); ^{13}C NMR (100 MHz, CD_3OD) δ 168.20, 143.19 (dm, *J* = 246.3 Hz), 141.91 (dm, *J* = 260.7 Hz), 139.41, 137.81, 134.09, 129.00, 125.81, 121.22, 118.48, 43.82, 34.02, 33.17, 32.86, 24.76, 24.39; HRMS (ESI+): calcd for $C_{21}H_{16}F_4N_2NaO$ $[M + Na]^+$ 411.10965, found 411.10968.

2-methyl-4-(perfluoropyridin-4-yl)benzo[*d*][1,3]oxazepine (**13**)

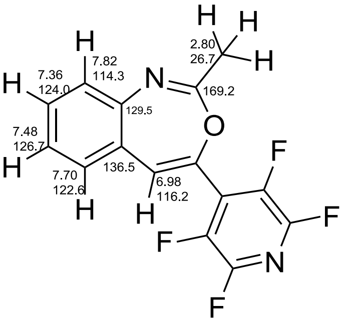

N-{2-[(Perfluoropyridin-4-yl)ethynyl]phenyl}acetamide **5** (5 mg, 0.016 mmol) and 1,4-cyclohexadiene (130 mg, 1.6 mmol) were dissolved in 1.6 ml of acetonitrile. After degassing by bubbling Ar into the solution for 10 minutes, the reaction mixture was irradiated under UV (310 nm) for 2.5 hours. The solvent was removed in vacuo and the product purified by column chromatograpy (EtOAc:hexane = 1:2): ^{1}H NMR (600 MHz, $CDCl_3$) δ 7.82 (d, *J* = 8.2 Hz, 1H), 7.70 (d, *J* = 7.8 Hz, 1H), 7.48 (ddd, *J* = 7.3 Hz, *J* = 7.3 Hz, *J* = 1.1 Hz, 1H), 7.36 (ddd, *J* = 7.7 Hz, *J* = 7.7 Hz, *J* = 0.5 Hz, 1H), 6.98 (s, 1H), 2.80 (s, 3H); ^{13}C NMR (150 MHz, $CDCl_3$) δ 169.1, 143.7 (dm, J = 243 Hz), 139.0 (dm, J = 257 Hz), 136.5, 129.5, 128.0 (m), 126.7, 124.0, 123.4, 122.6, 116.2, 114.3, 26.6; HRMS (EI+): calcd for $C_{15}H_8F_4N_2O$ $[M]^+$ 308.05728, found 308.05727; m.p. 98-99 °C.

***N*-{2-[2-(perfluoropyridin-4-yl)acetyl]phenyl}acetamide** (**14**)

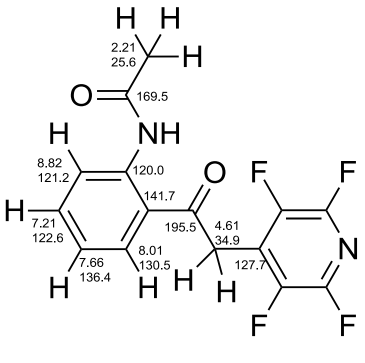

N{(2-[(Perfluoropyridin-4-yl)ethyny]phenyl}acetamide **5** (5 mg, 0.016 mmol) and 1,4-cyclohexadiene (130 mg, 1.6 mmol) were dissolved in 1.6 ml of acetonitrile. After degassing by bubbling Ar into the solution for 10 minutes, the reaction mixture was irradiated under UV (310 nm) for 2.5 hours. The solvent was removed in vacuo and the product was purified by column chromatograpy (EtOAc:hexane = 1:2): ^{1}H NMR (400 MHz,

$CDCl_3$) δ 11.28 (s, 1H), 8.82 (d, *J* = 8.6 Hz, 1H), 8.01 (d, *J* = 7.6 Hz, 1H), 7.66 (dd, *J* = 8.2 Hz, *J* = 8.2 Hz, 1H), 7.21 (dd, *J* = 7.8 Hz, *J* = 7.8 Hz, 1H), 4.61 (s, 2H), 2.21 (s, 3H); ^{13}C NMR (100 MHz, $CDCl_3$) δ 195.5, 169.5, 143.2 (dm, *J* = 258 Hz), 141.7, 140.9 (dm, *J* = 241 Hz), 136.4, 130.5, 127.7 (m), 122.6, 121.2, 120.0, 34.9, 25.6; HRMS (ESI+): calcd for $C_{15}H_{10}F_4N_2NaO_2$ [M + Na]$^+$ 349.05761, found 349.05787; m.p. 163-164 °C.

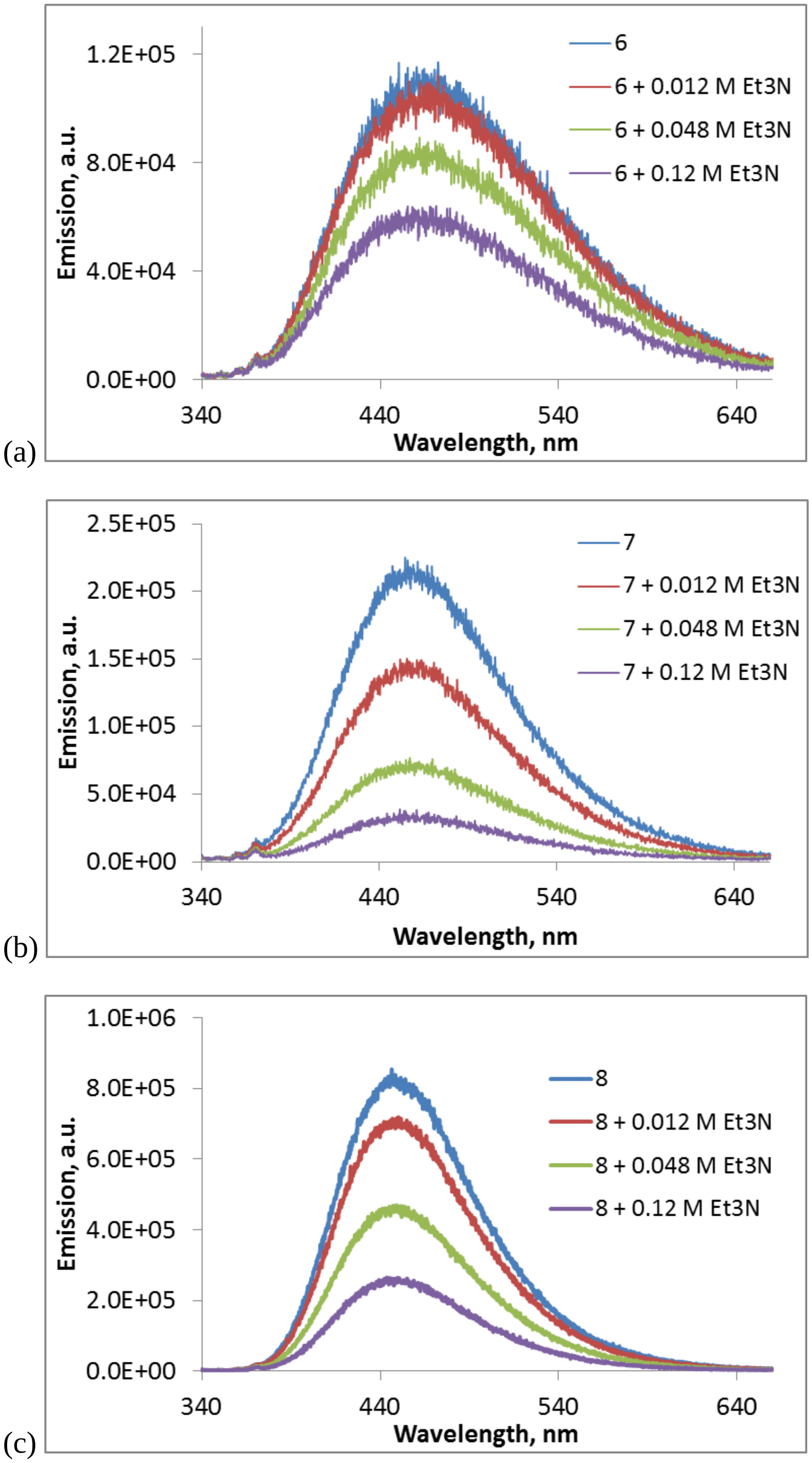

Figure S1: Emission titration of **6** (a), **7** (b) and **8** (c) in acetonitrile (10 μM) with 0, 0.012, 0.048 and 0.12 M of Et_3N. Excitation : 330 nm.

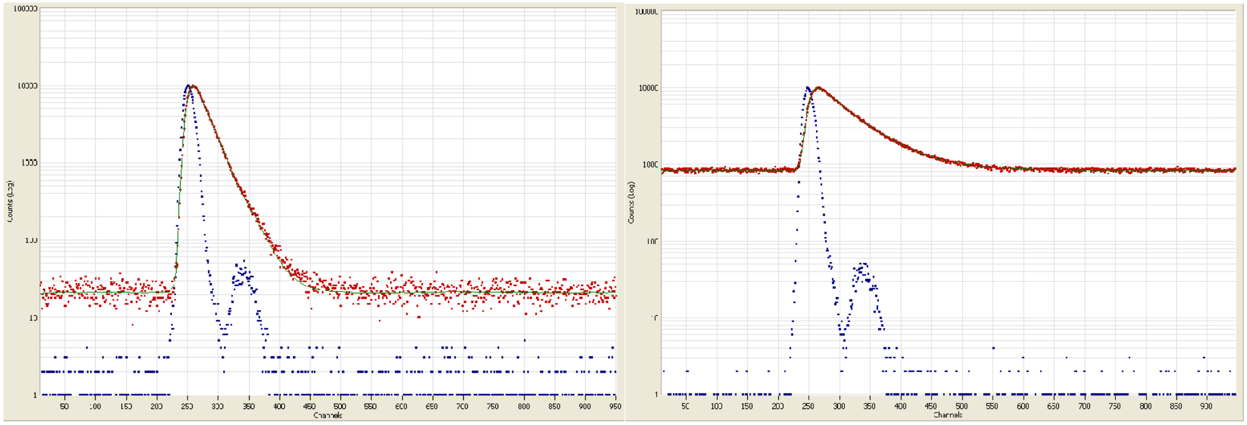

(a) (b)

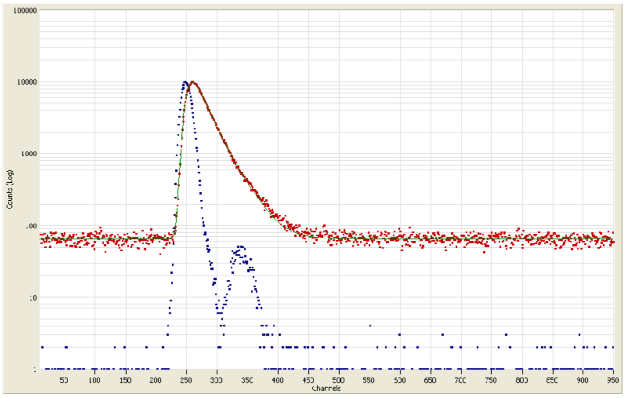

(c)

Figure S2: The fluorescence decay traces of **3** (a), **4** (b) and **5** (c) in acetonitrile.

Table S1: Analyzed fluorescence decay data for three isomers **3**, **4** and **5**.

Compound	lifetime (τ)	s. dev.	χ^2
**3**	1.26×10^{-9}	3.22×10^{-12}	1.31
**4**	3.35×10^{-9}	9.30×10^{-12}	1.54
**5**	1.34×10^{-9}	3.54×10^{-12}	1.38

compound	**7**	**7**	**7**	**7**	**6**	**6**	**6**	**6**	**1**	**1**	**1**	**1**
pH	6	6	7	8	6	6	7	8	6	6	7	8
UV	-	+	+	+	-	+	+	+	-	+	+	+

Form II

Form III

Form I

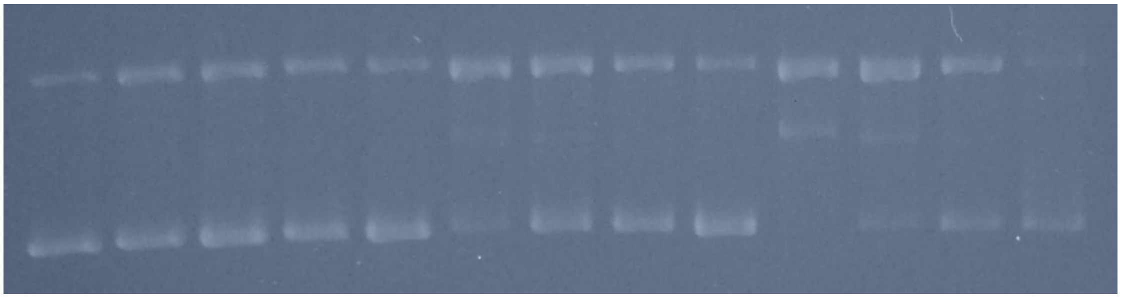

Figure S3: Picture of plasmid relaxation assay with 15 μM of lysine conjugates, **1**, **6** and **7,** and pBR 322 plasmid DNA (30 μM/bp) in 20 mM sodium phosphate buffer after 10 min of irradiation. Form I: intact supercoiled DNA, form II: relaxed form (ss cleavage), form III: linear form (ds cleavage).

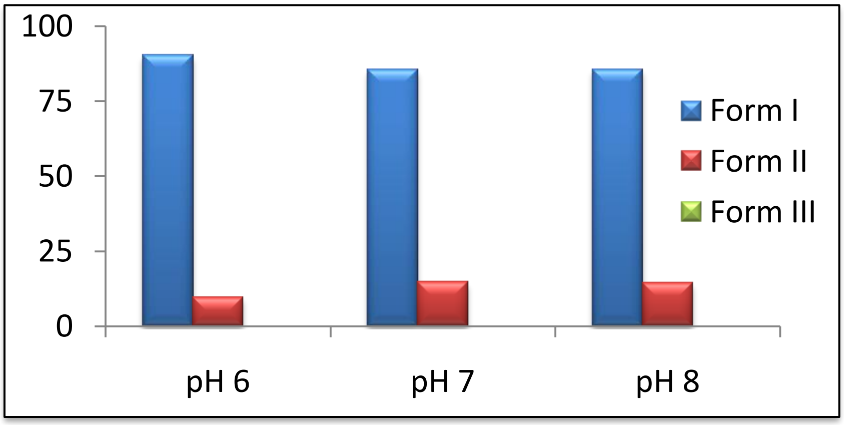

Figure S4: Quantitative plots of plasmid relaxation assays with 30 μM (b.p.) of pBR 322 after 10 min. of UV (>300 nm wavelength) irradiation at pH 6, 7 and 8.

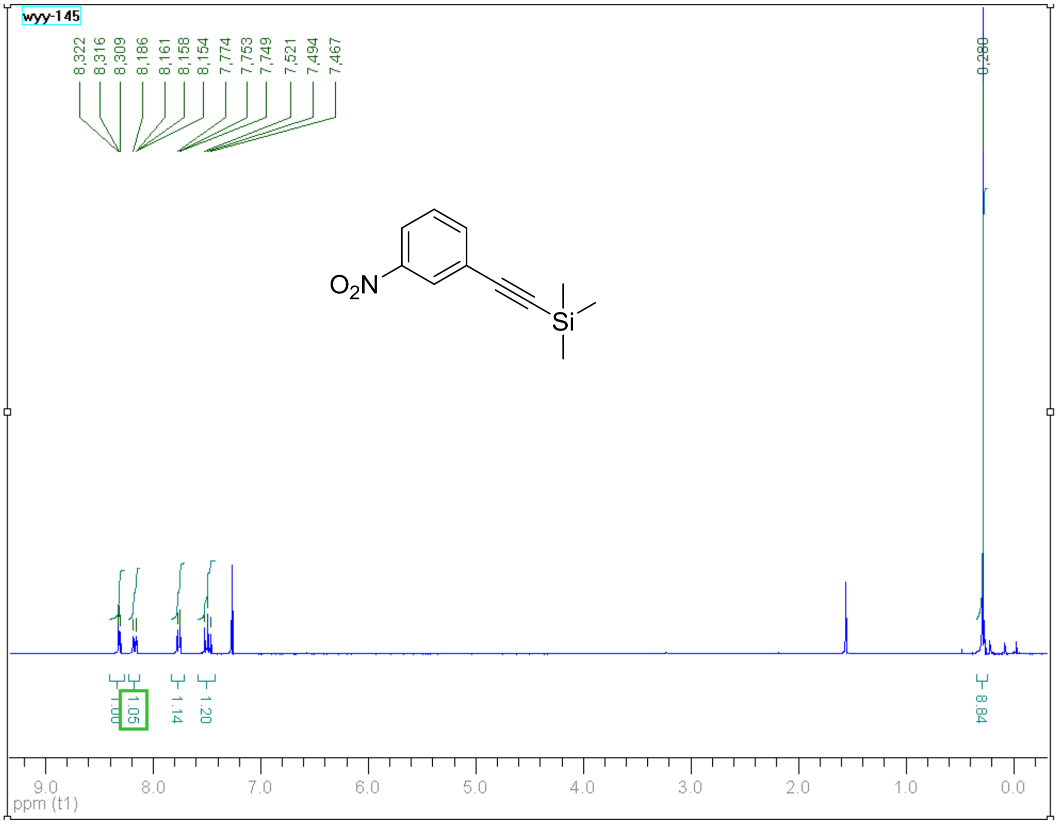

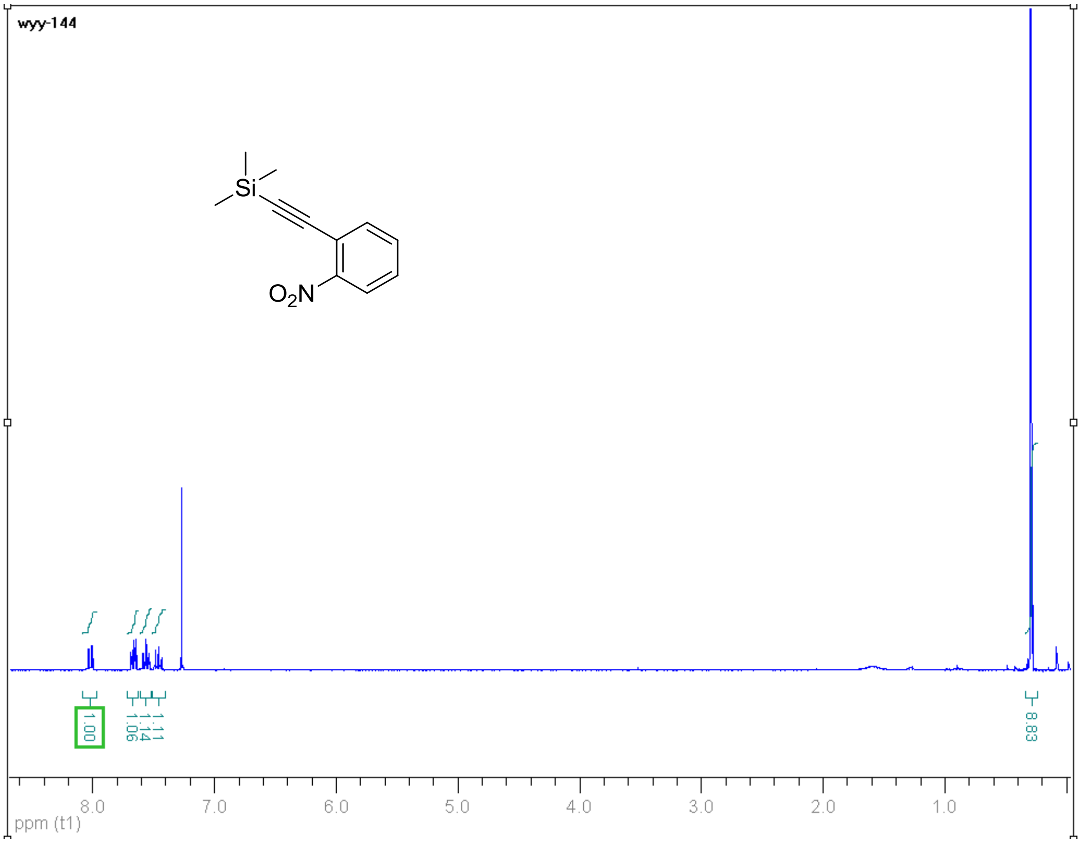

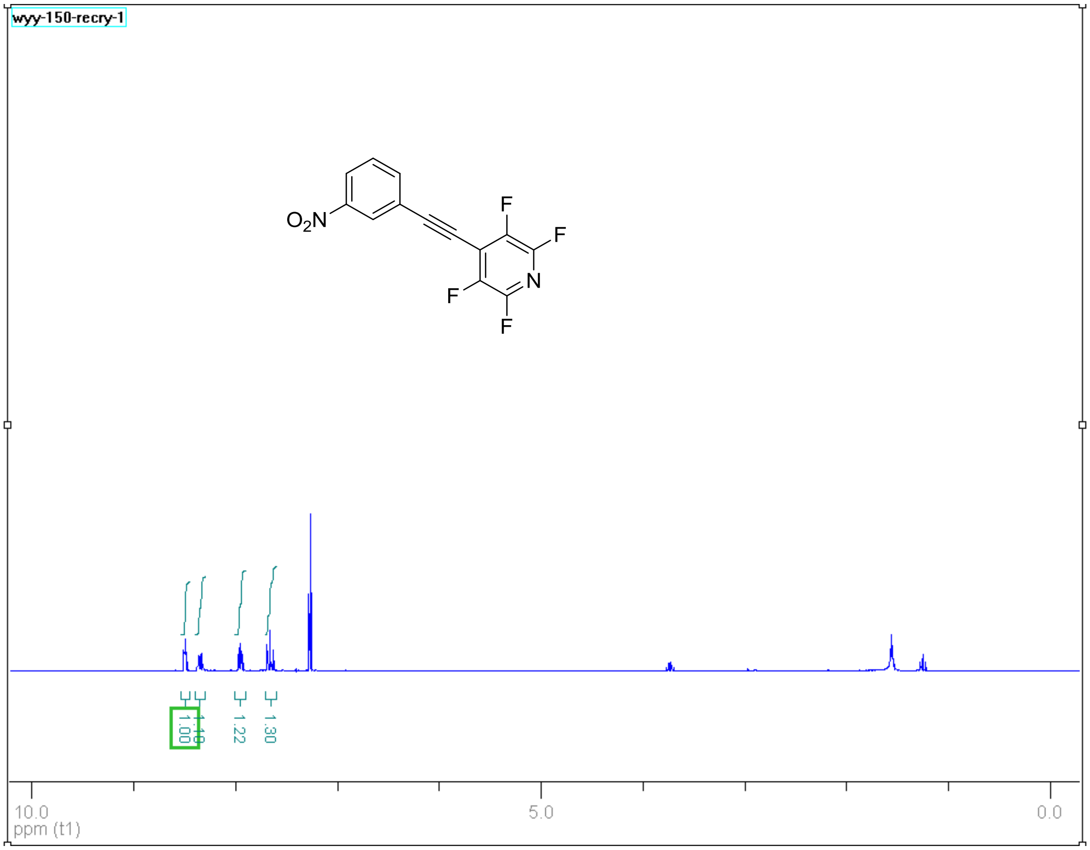
wyy-150-recry-1
O_2N
F
F
N
F
F
1.00
1.18
1.22
1.30
10.0
5.0
0.0
ppm (t1)

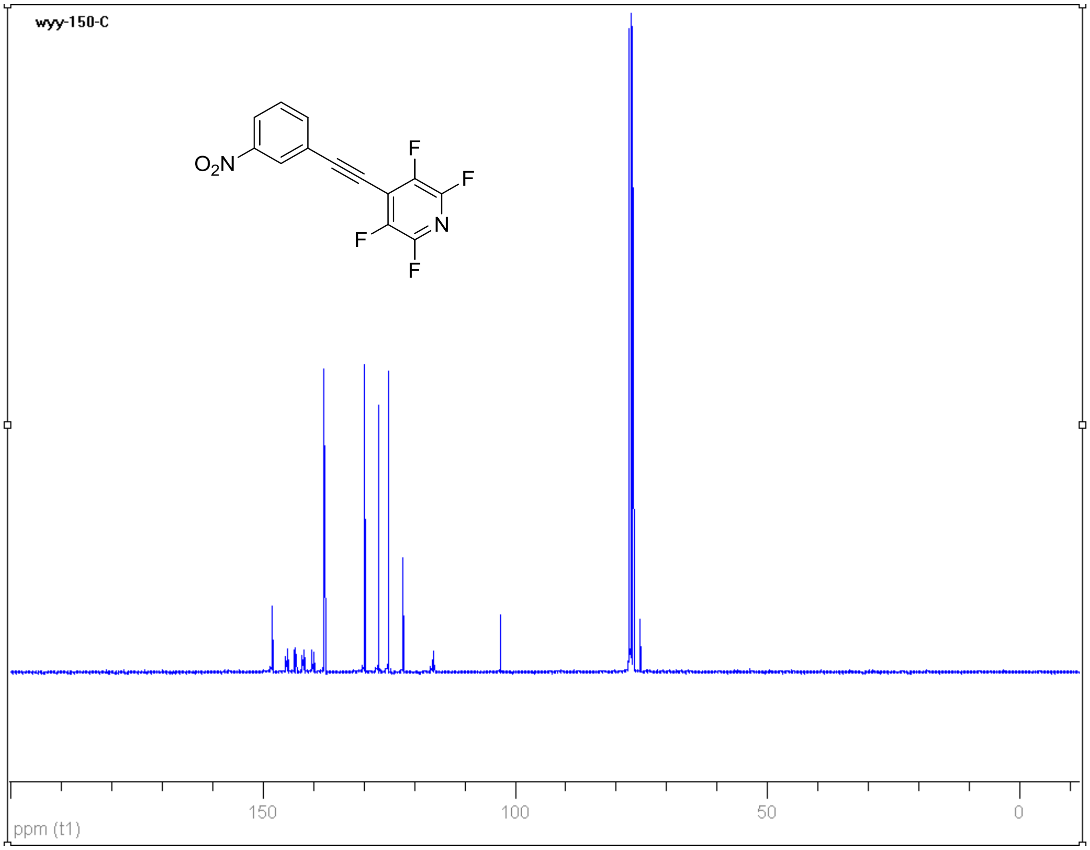
wyy-150-C
O_2N
F
F
N
F
F
150
100
50
0
ppm (t1)

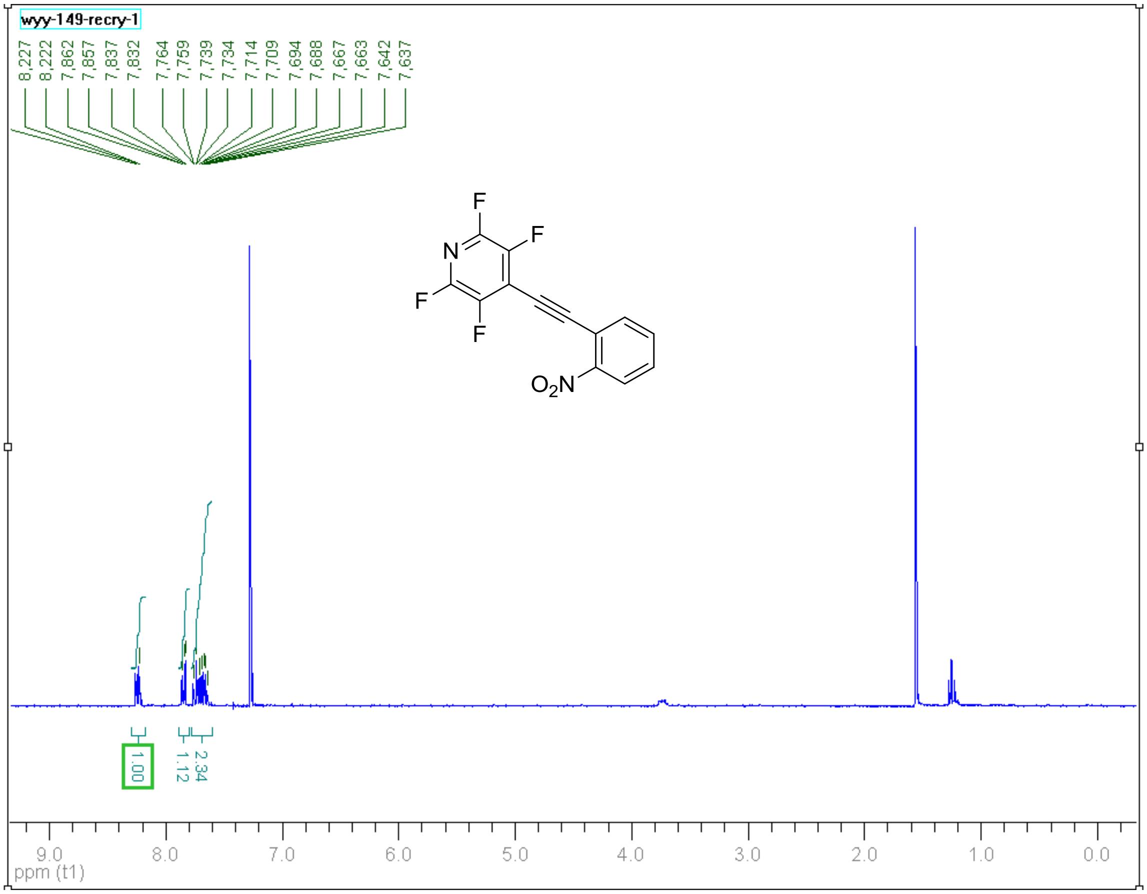
wyy-149-recry-1
8.227
8.222
7.862
7.857
7.837
7.832
7.764
7.759
7.739
7.734
7.714
7.709
7.694
7.688
7.667
7.663
7.642
7.637
F
F
N
F
F
O2N
1.00
1.12
2.34
9.0
8.0
7.0
6.0
5.0
4.0
3.0
2.0
1.0
0.0
ppm (t1)

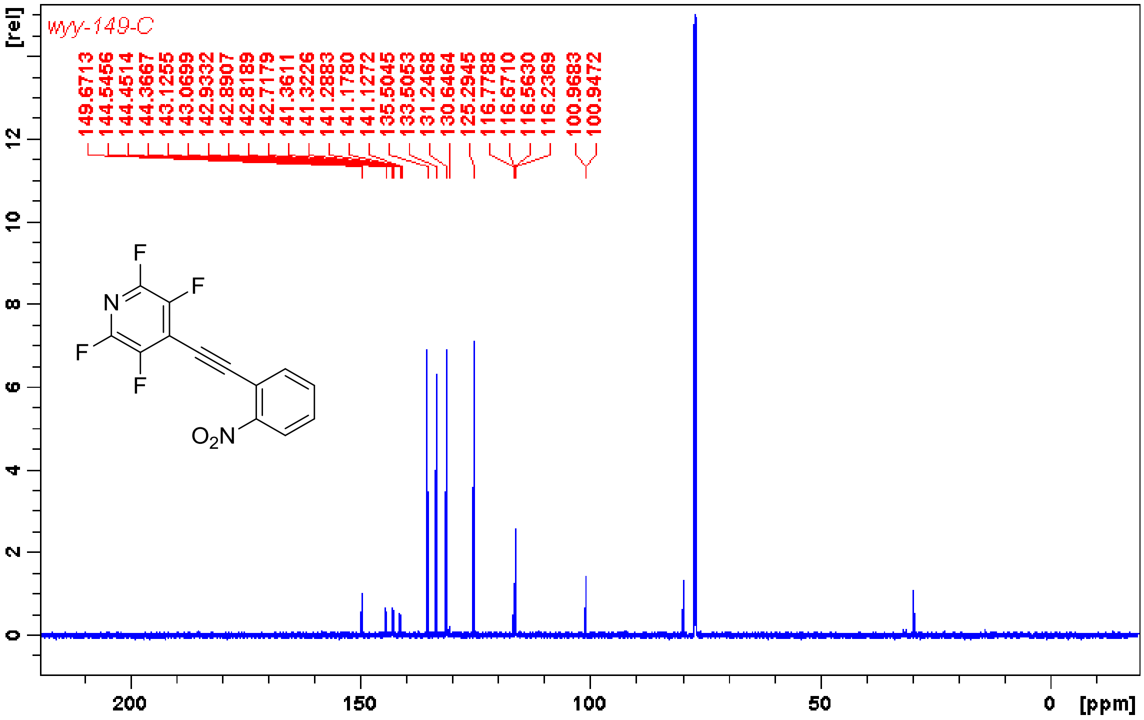
wyy-149-C
149.6713
144.5456
144.4514
144.3667
143.1255
143.0699
142.9332
142.8907
142.8189
142.7179
141.3611
141.3226
141.2883
141.1780
141.1272
135.5045
133.5053
131.2468
130.6464
125.2945
116.7788
116.6710
116.5630
116.2369
100.9683
100.9472
[rel]
F
F
N
F
F
O2N
200
150
100
50
0
[ppm]

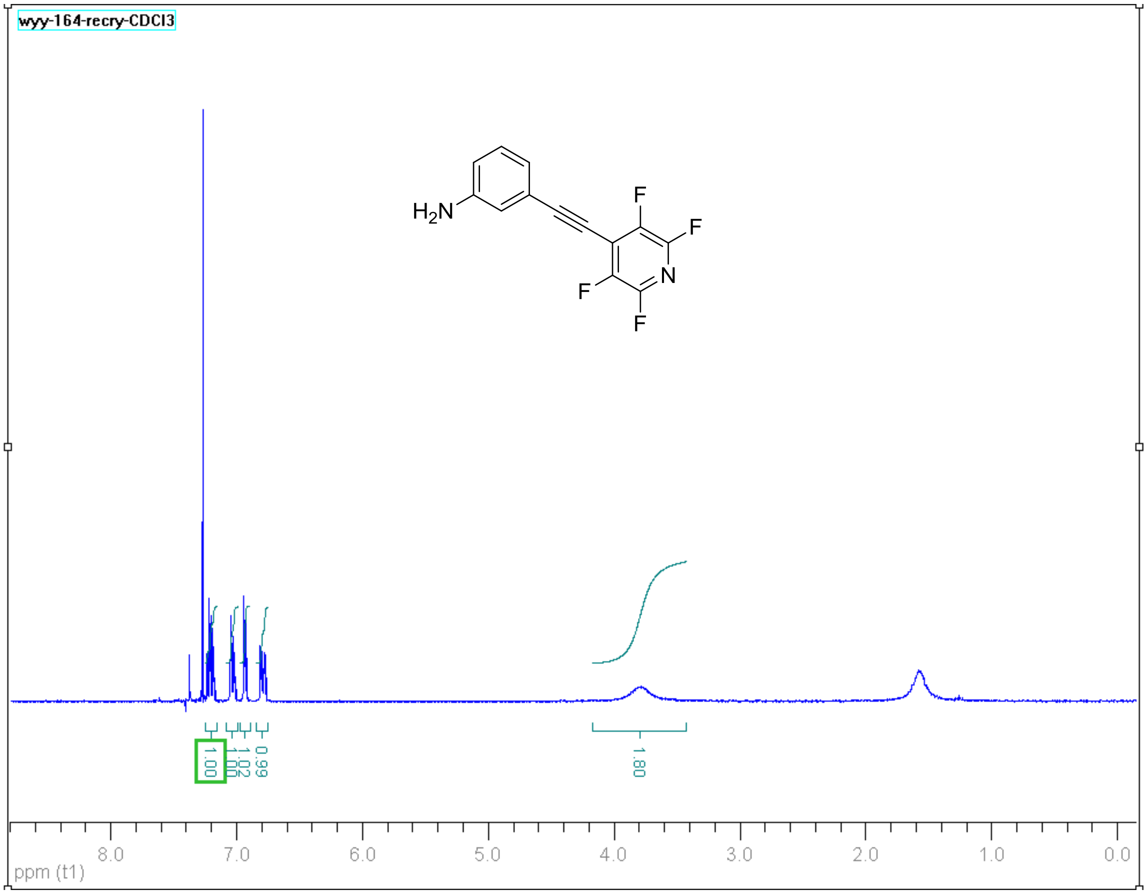
wyy-164-recry-CDCl3
H_2N
F
F
F
F
N
1.00
1.06
1.02
0.99
1.80
8.0
7.0
6.0
5.0
4.0
3.0
2.0
1.0
0.0
ppm (t1)

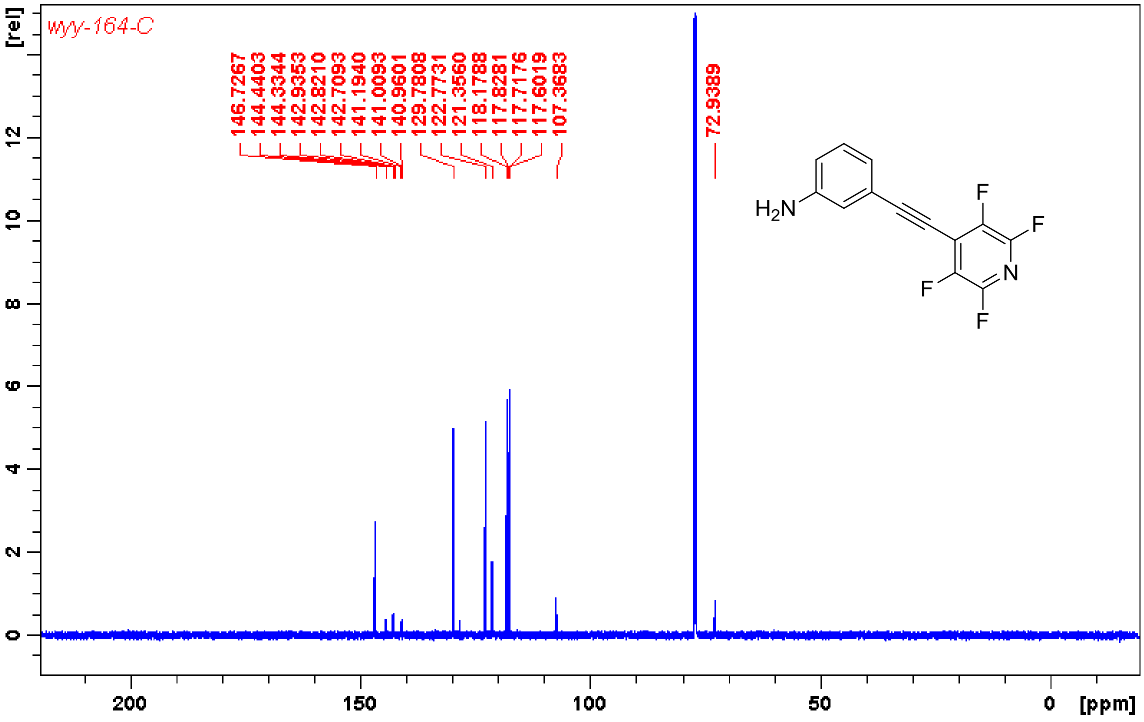
wyy-164-C
146.7267
144.4403
144.3344
142.9353
142.8210
142.7093
141.1940
141.0093
140.9601
129.7808
122.7731
121.3560
118.1788
117.8281
117.7176
117.6019
107.3683
72.9389
H_2N
F
F
F
F
N
[rel]
200
150
100
50
0
[ppm]

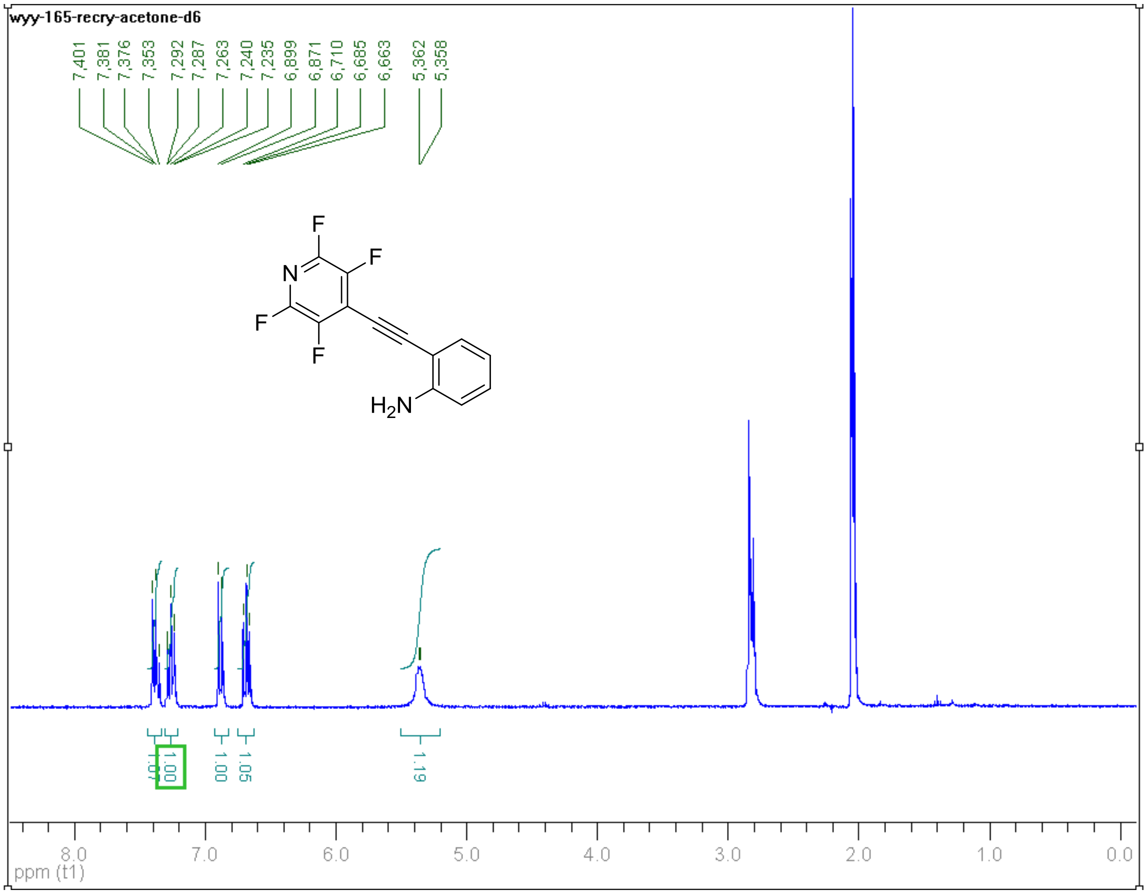
wyy-165-recry-acetone-d6
7.401
7.381
7.376
7.353
7.292
7.287
7.263
7.240
7.235
6.899
6.871
6.710
6.685
6.663
5.362
5.358
F
F
N
F
F
H2N
1.00
1.00
1.05
1.19
8.0
7.0
6.0
5.0
4.0
3.0
2.0
1.0
0.0
ppm (t1)

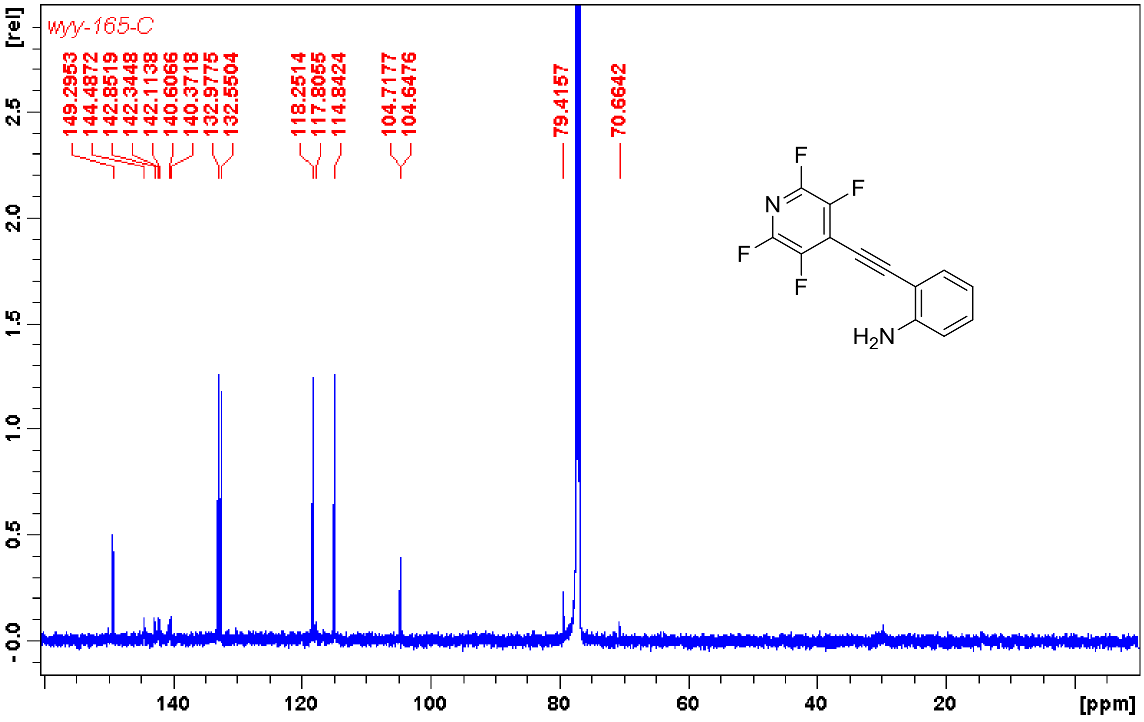
[rel]
wyy-165-C
149.2953
144.4872
142.8519
142.3448
142.1138
140.6066
140.3718
132.9775
132.5504
118.2514
117.8055
114.8424
104.7177
104.6476
79.4157
70.6642
F
F
N
F
F
H2N
2.5
2.0
1.5
1.0
0.5
-0.0
140
120
100
80
60
40
20
[ppm]

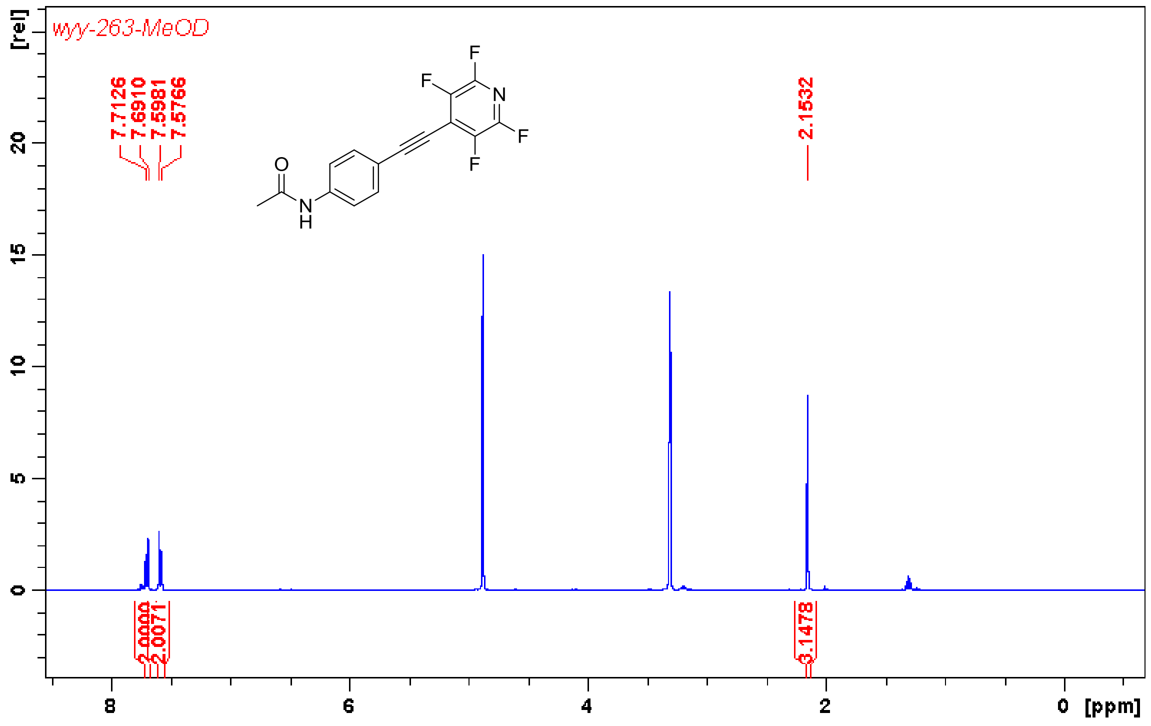
wyy-263-MeOD
7.7126
7.6910
7.5981
7.5766
2.1532
2.0000
2.0071
3.1478
[rel]
[ppm]

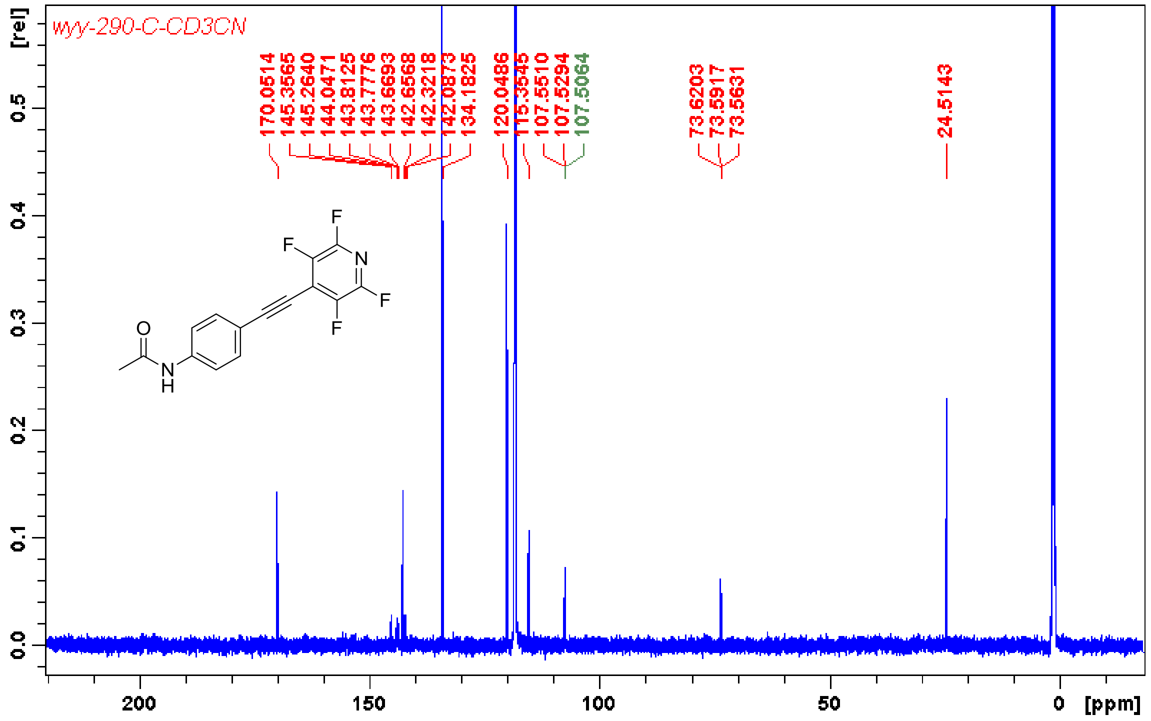
wyy-290-C-CD3CN
170.0514
145.3565
145.2640
144.0471
143.8125
143.7776
143.6693
142.6568
142.3218
142.0873
134.1825
120.0486
115.3545
107.5510
107.5294
107.5064
73.6203
73.5917
73.5631
24.5143
[rel]
[ppm]

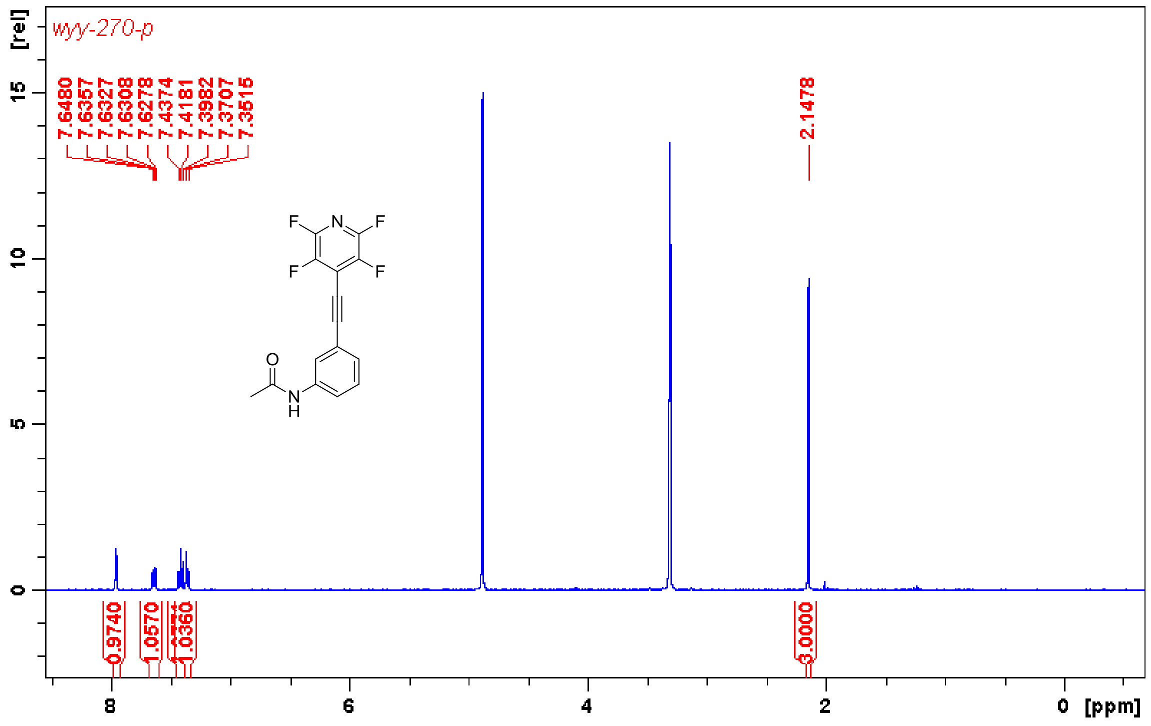
wyy-270-p
7.6480
7.6357
7.6327
7.6308
7.6278
7.4374
7.4181
7.3982
7.3707
7.3515
2.1478
0.9740
1.0570
1.0360
3.0000
[rel]
[ppm]

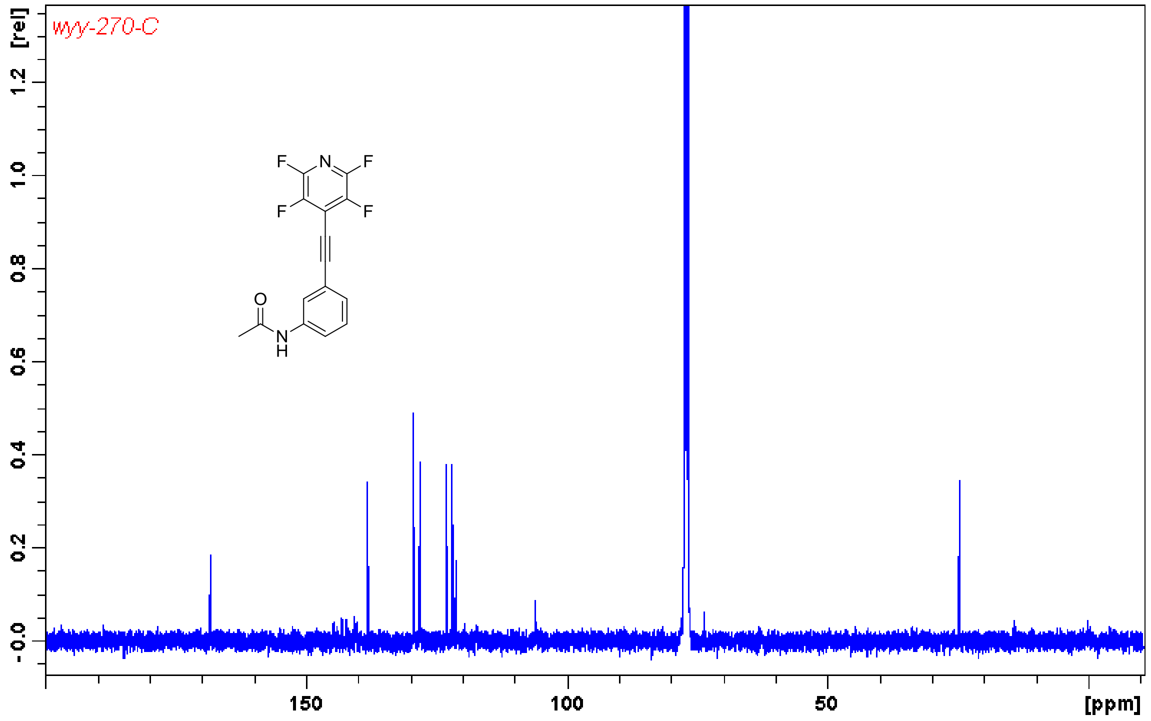
wyy-270-C
[rel]
[ppm]

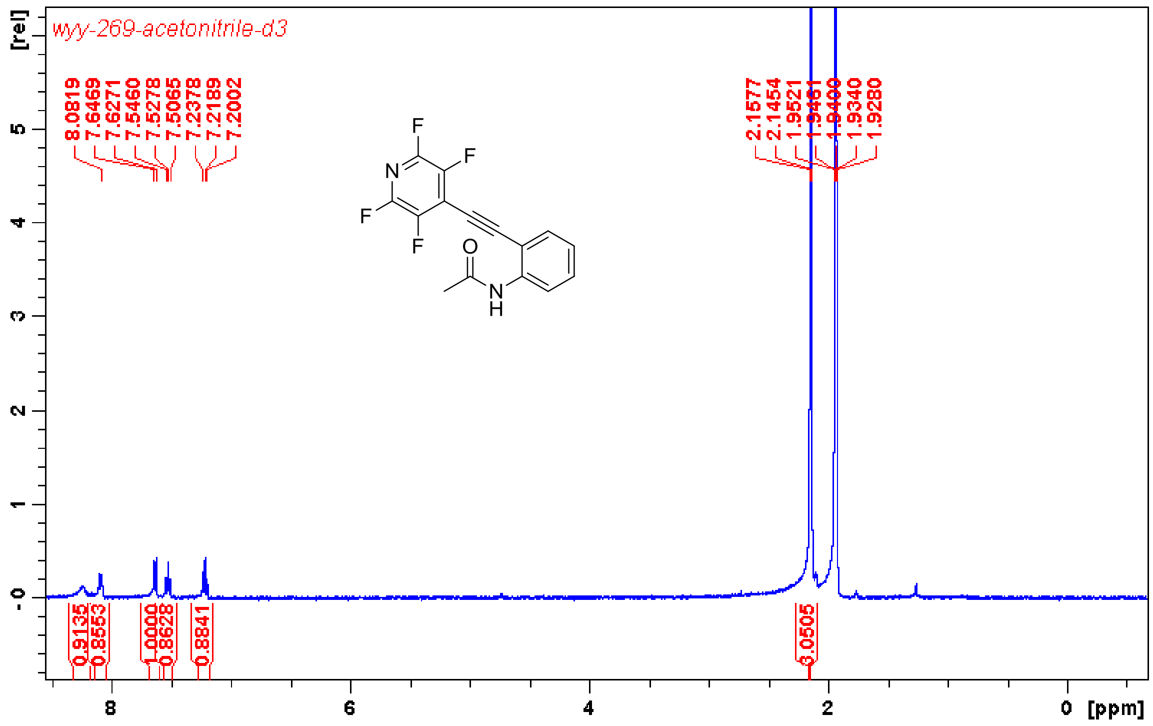
wyy-269-acetonitrile-d3
8.0819
7.6469
7.6271
7.5460
7.5278
7.5065
7.2378
7.2189
7.2002
2.1577
2.1454
1.9521
1.9461
1.9400
1.9340
1.9280
0.9135
0.8553
1.0000
0.8628
0.8841
3.0505
[rel]
[ppm]
F
N
H
O

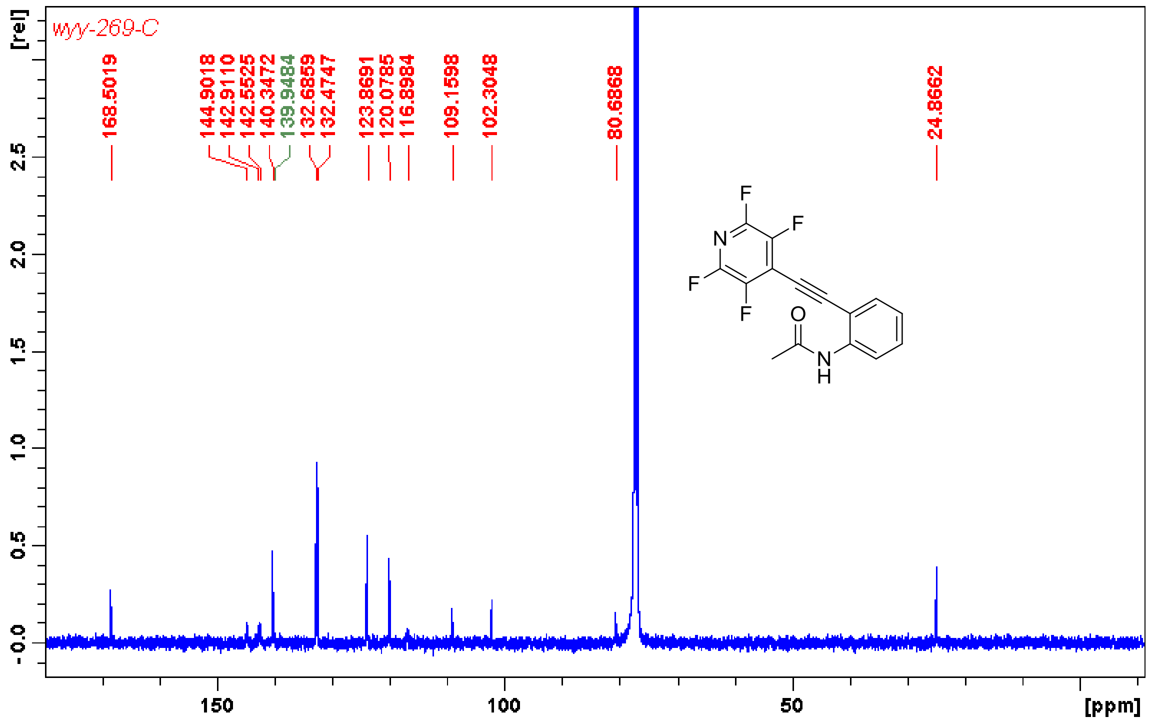
wyy-269-C
168.5019
144.9018
142.9110
142.5525
140.3472
139.9484
132.6859
132.4747
123.8691
120.0785
116.8984
109.1598
102.3048
80.6868
24.8662
[rel]
[ppm]
F
N
H
O

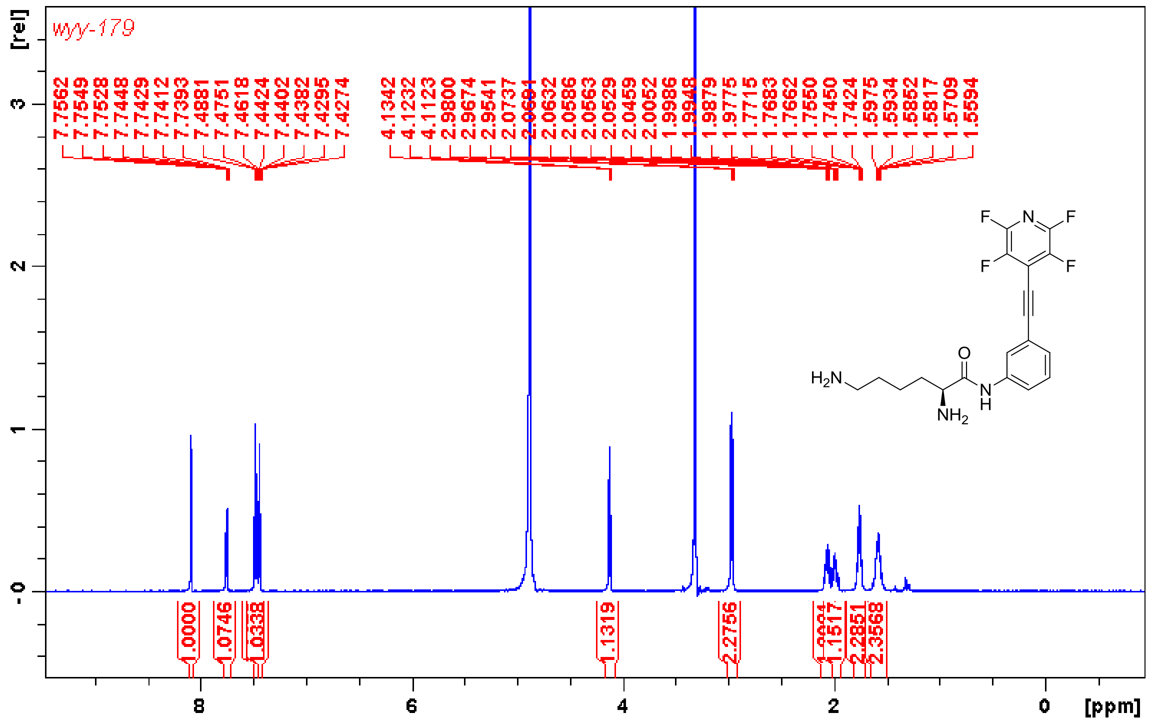
wyy-179
[rel]
[ppm]
H2N
NH2
N
H
O
F
N
F
F
F

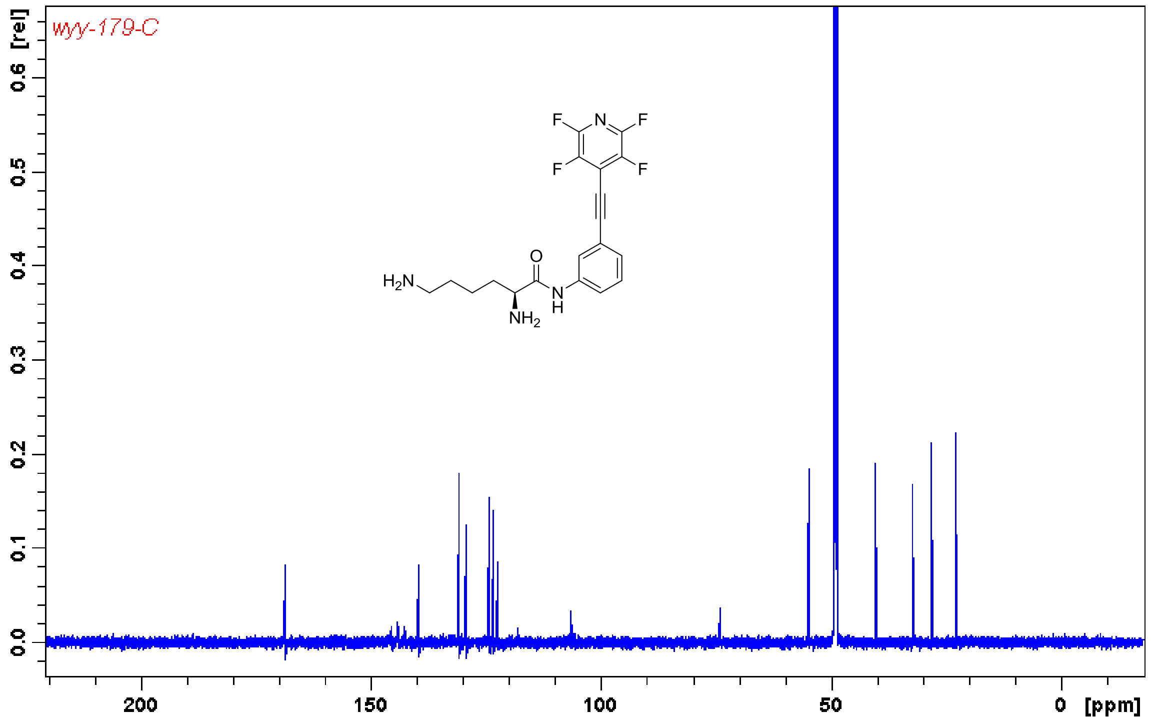
wyy-179-C
[rel]
[ppm]
H2N
NH2
N
H
O
F
N
F
F
F

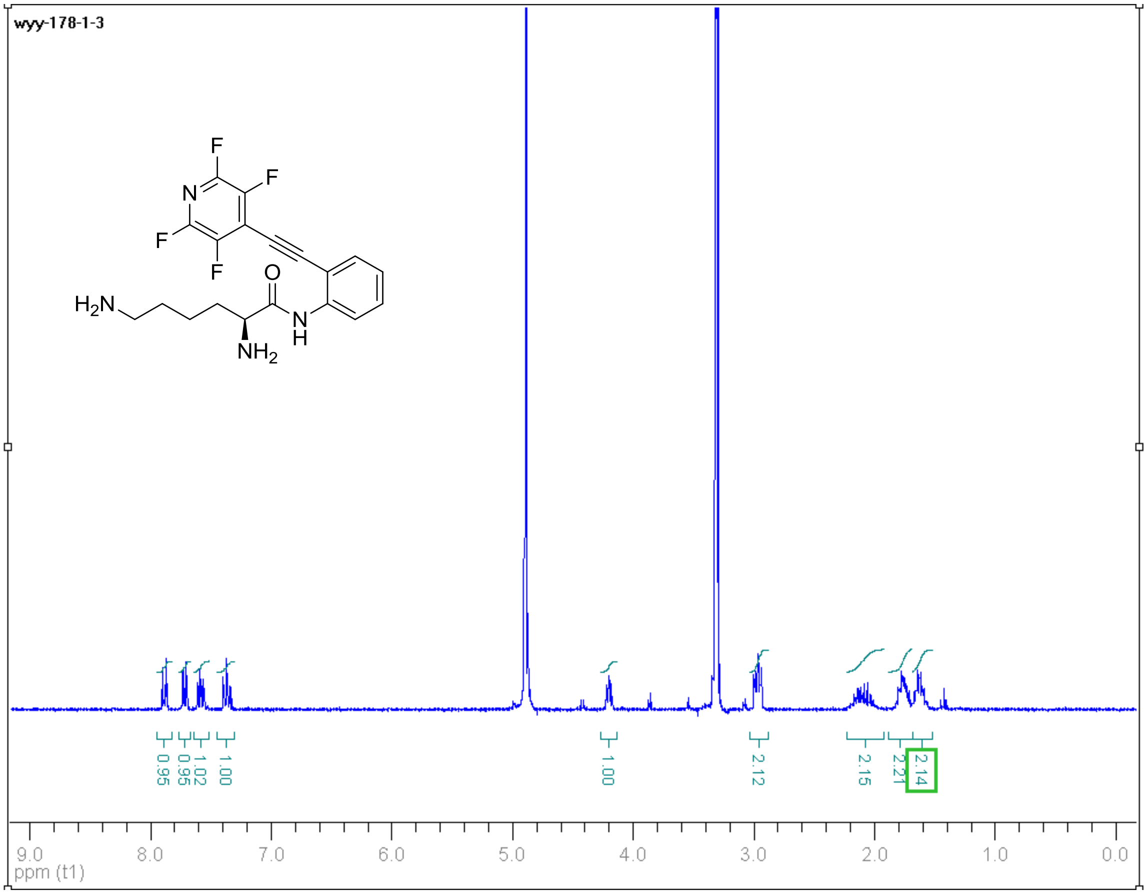
wyy-178-1-3
F
F
N
F
F
O
H2N
N
H
NH2
0.95
0.95
1.02
1.00
1.00
2.12
2.15
2.21
2.14
9.0
8.0
7.0
6.0
5.0
4.0
3.0
2.0
1.0
0.0
ppm (t1)

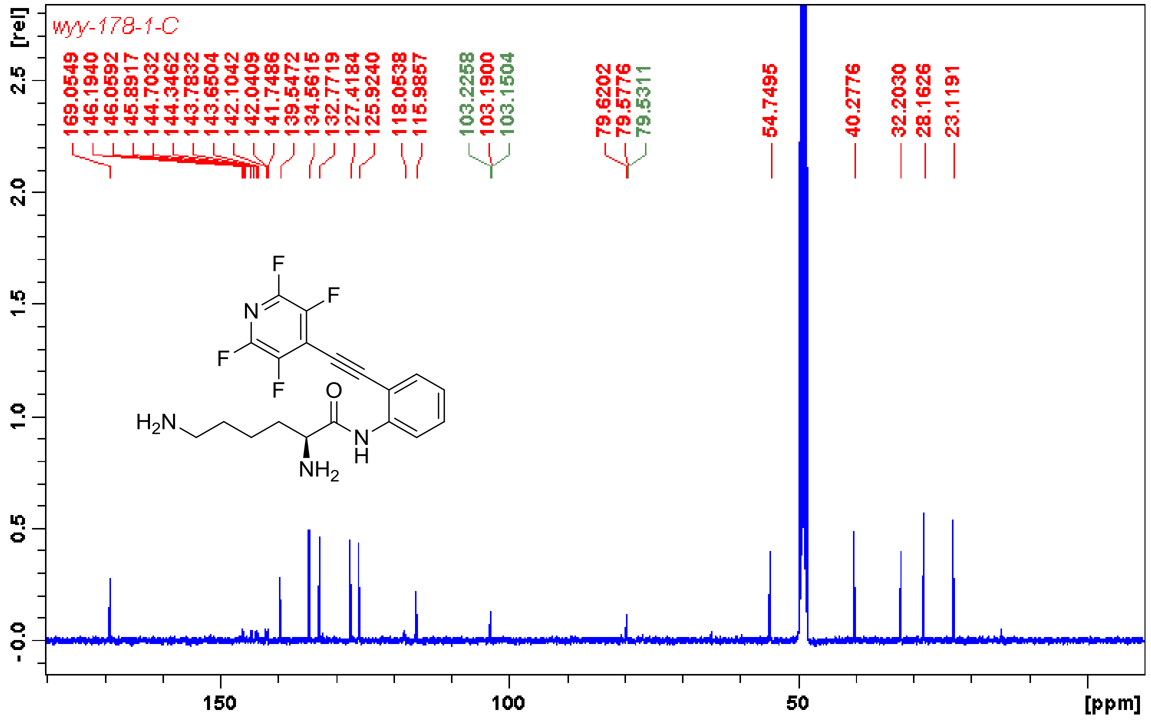
wyy-178-1-C
169.0549
146.1940
146.0592
145.8917
144.7032
144.3462
143.7832
143.6504
142.1042
142.0409
141.7486
139.5472
134.5615
132.7719
127.4184
125.9240
118.0538
115.9857
103.2258
103.1900
103.1504
79.6202
79.5776
79.5311
54.7495
40.2776
32.2030
28.1626
23.1191
[rel]
2.5
2.0
1.5
1.0
0.5
- 0.0
150
100
50
[ppm]
F
F
N
F
F
O
H2N
N
H
NH2

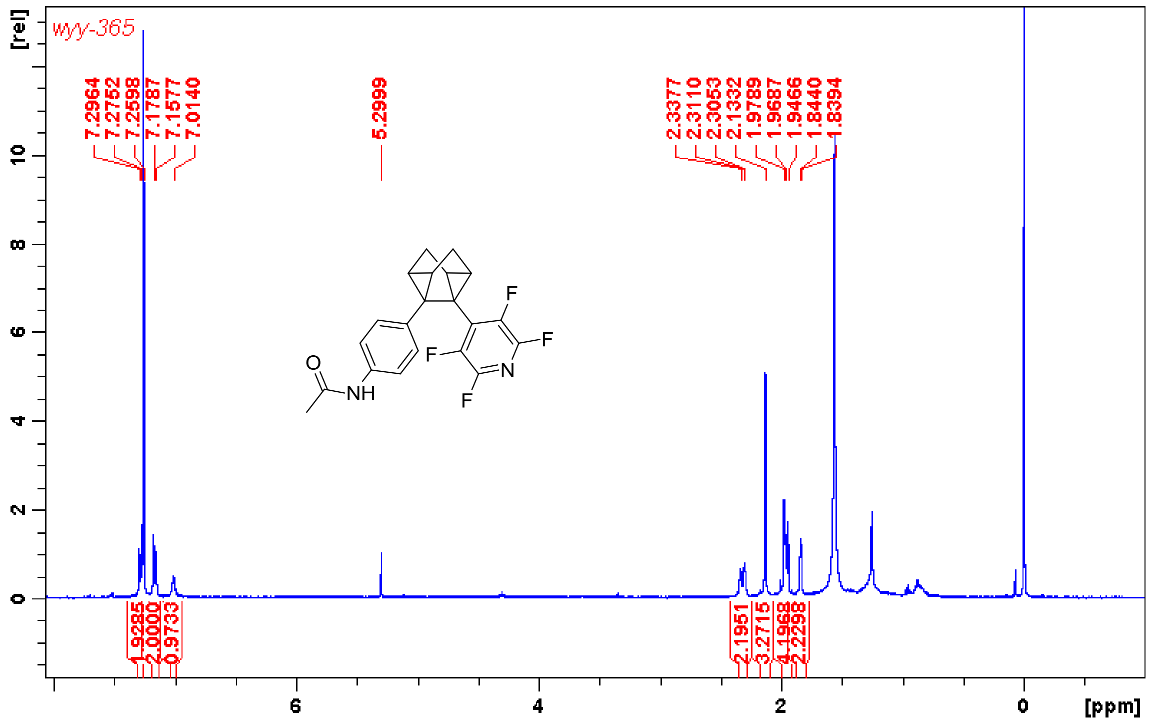
wyy-365
7.2964
7.2752
7.2598
7.1787
7.1577
7.0140
5.2999
2.3377
2.3110
2.3053
2.1332
1.9789
1.9687
1.9466
1.8440
1.8394
1.9285
2.0000
0.9733
2.1951
3.2715
4.1968
2.2298
O
NH
F
F
F
F
N
[rel]
[ppm]

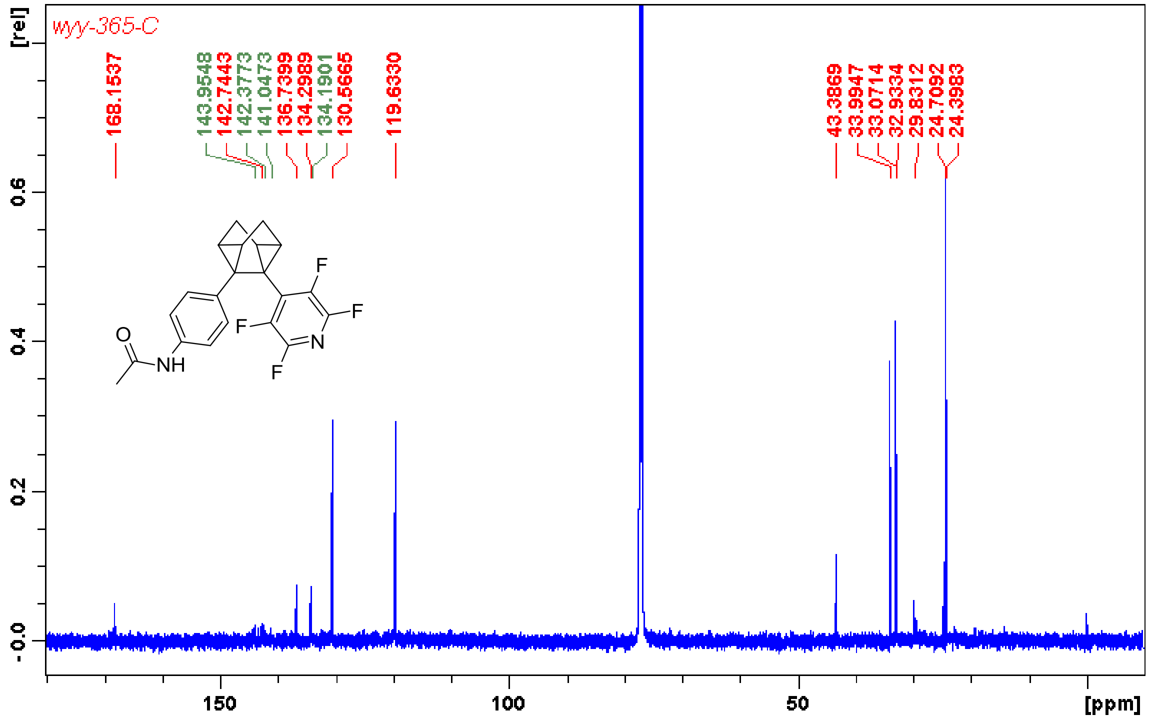
wyy-365-C
168.1537
143.9548
142.7443
142.3773
141.0473
136.7399
134.2989
134.1901
130.5665
119.6330
43.3869
33.9947
33.0714
32.9334
29.8312
24.7092
24.3983
O
NH
F
F
F
F
N
[rel]
[ppm]

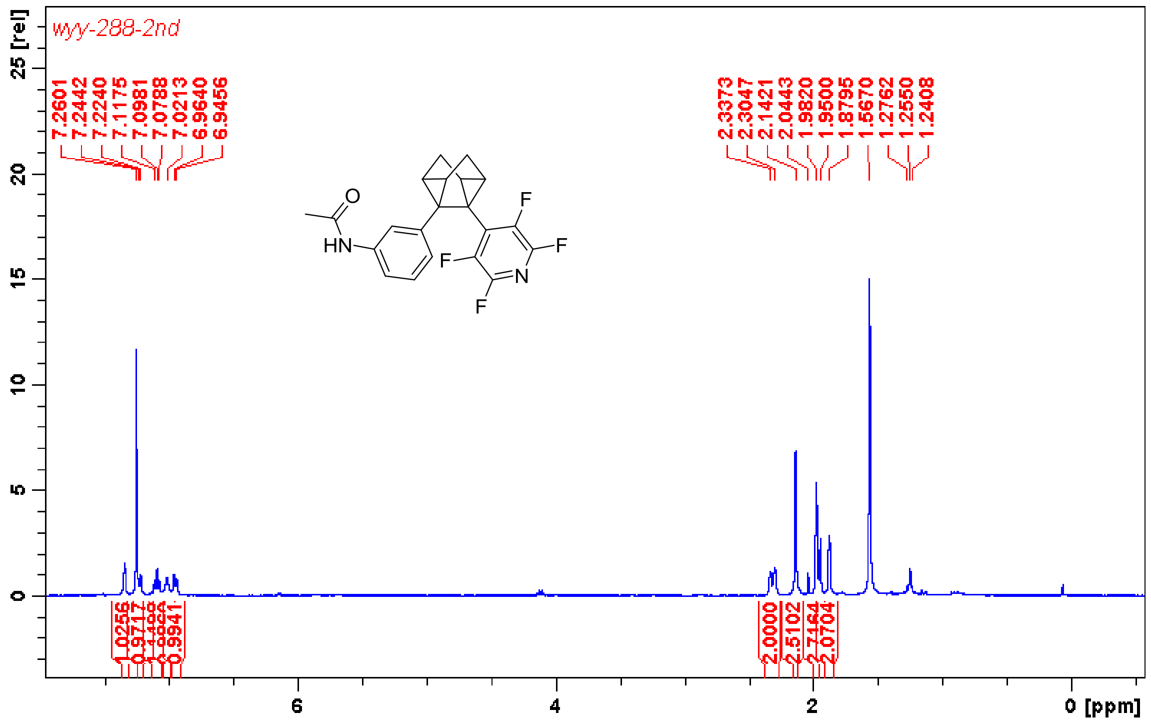
wyy-288-2nd
7.2601
7.2442
7.2240
7.1175
7.0981
7.0788
7.0213
6.9640
6.9456
2.3373
2.3047
2.1421
2.0443
1.9820
1.9500
1.8795
1.5670
1.2762
1.2550
1.2408
1.0256
0.9717
0.9941
2.0000
2.5102
2.0704
HN
O
F
F
F
F
N
[ppm]
[rel]

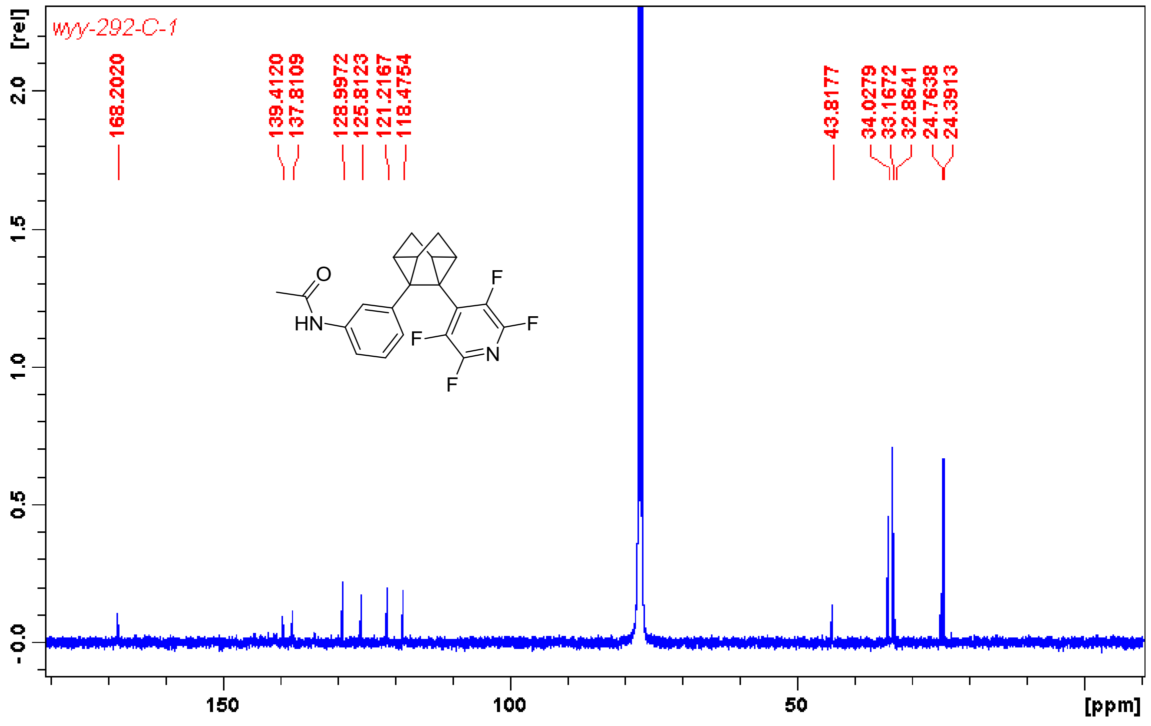
wyy-292-C-1
168.2020
139.4120
137.8109
128.9972
125.8123
121.2167
118.4754
43.8177
34.0279
33.1672
32.8641
24.7638
24.3913
HN
O
F
F
F
F
N
[ppm]
[rel]

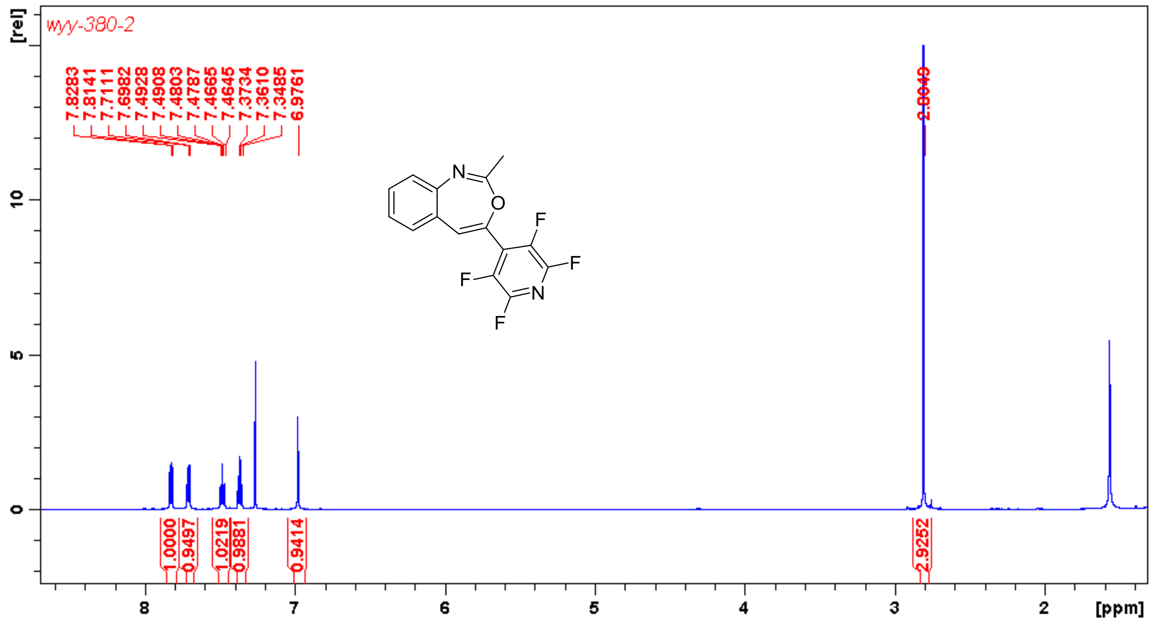
wyy-380-2
7.8283
7.8141
7.7111
7.6982
7.4928
7.4908
7.4803
7.4787
7.4665
7.4645
7.3734
7.3610
7.3485
6.9761
2.8049
1.0000
0.9497
1.0219
0.9881
0.9414
2.9252
[rel]
[ppm]

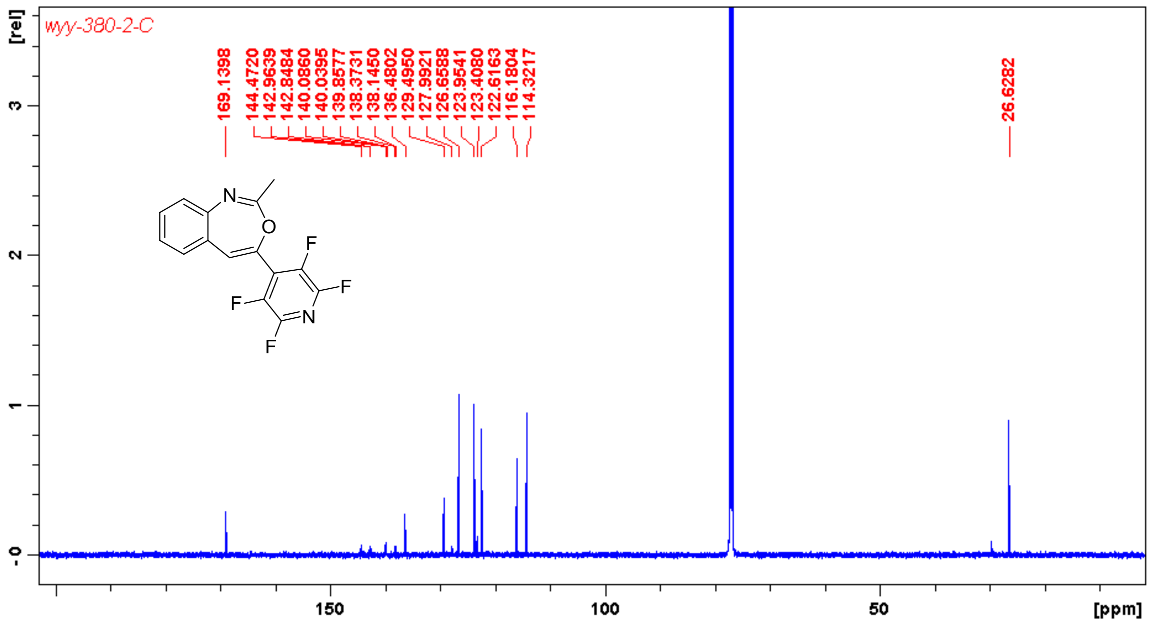
wyy-380-2-C
169.1398
144.4720
142.9639
142.8484
140.0860
140.0395
139.8577
138.3731
138.1450
136.4802
129.4950
127.9921
126.6588
123.9541
123.4080
122.6163
116.1804
114.3217
26.6282
[rel]
[ppm]

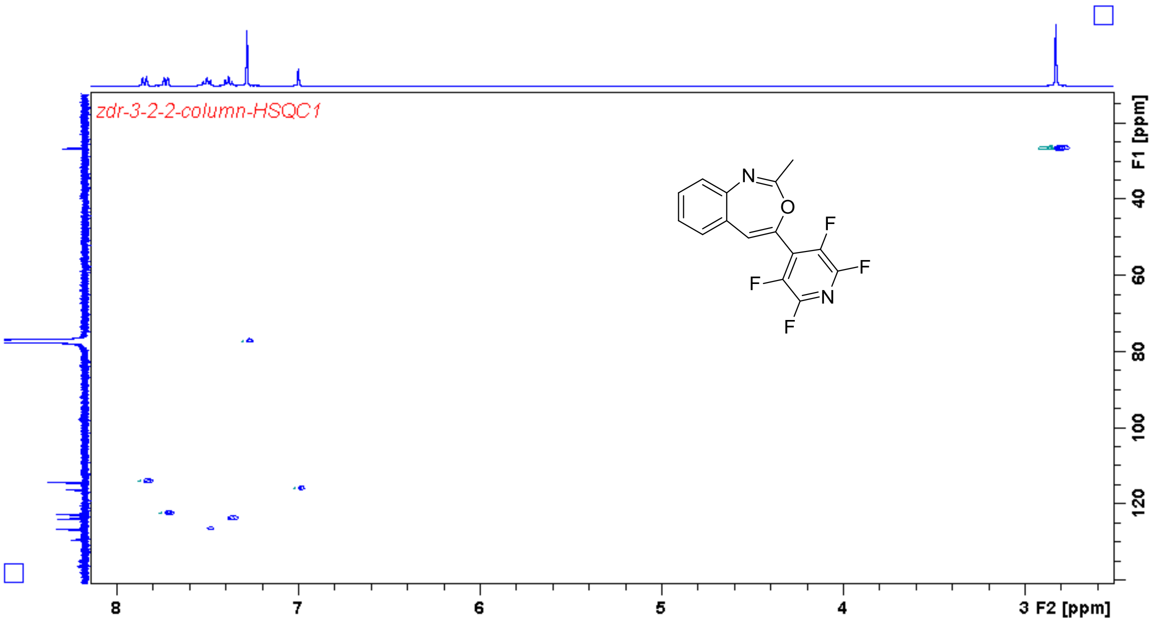
zdr-3-2-2-column-HSQC1
F1 [ppm]
F2 [ppm]

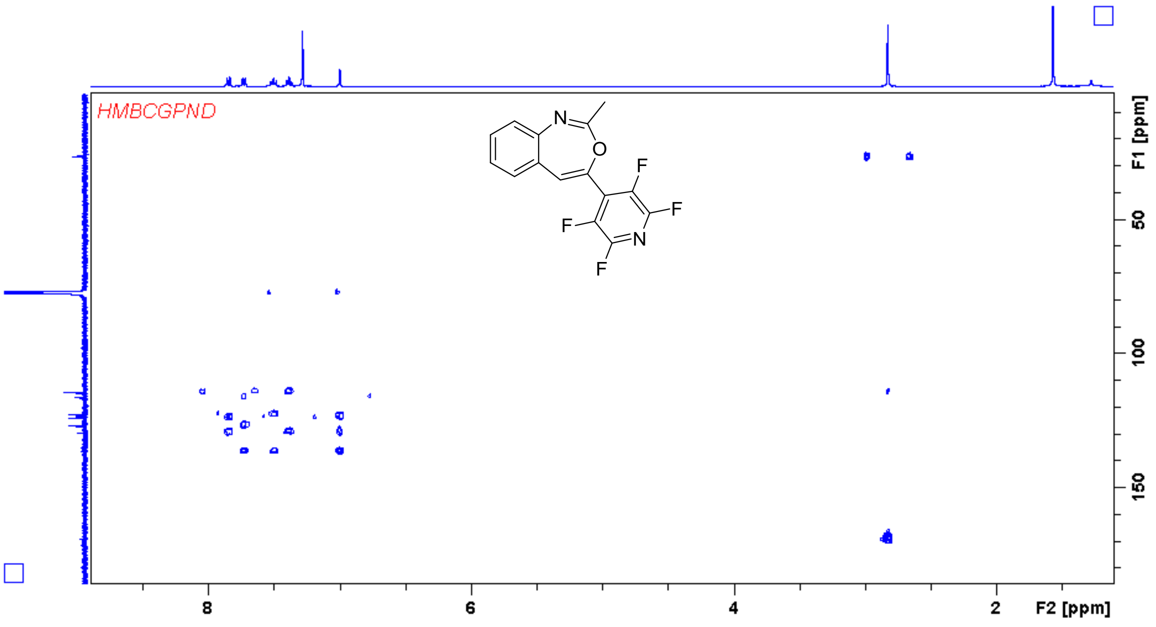
HMBCGPND
F1 [ppm]
F2 [ppm]

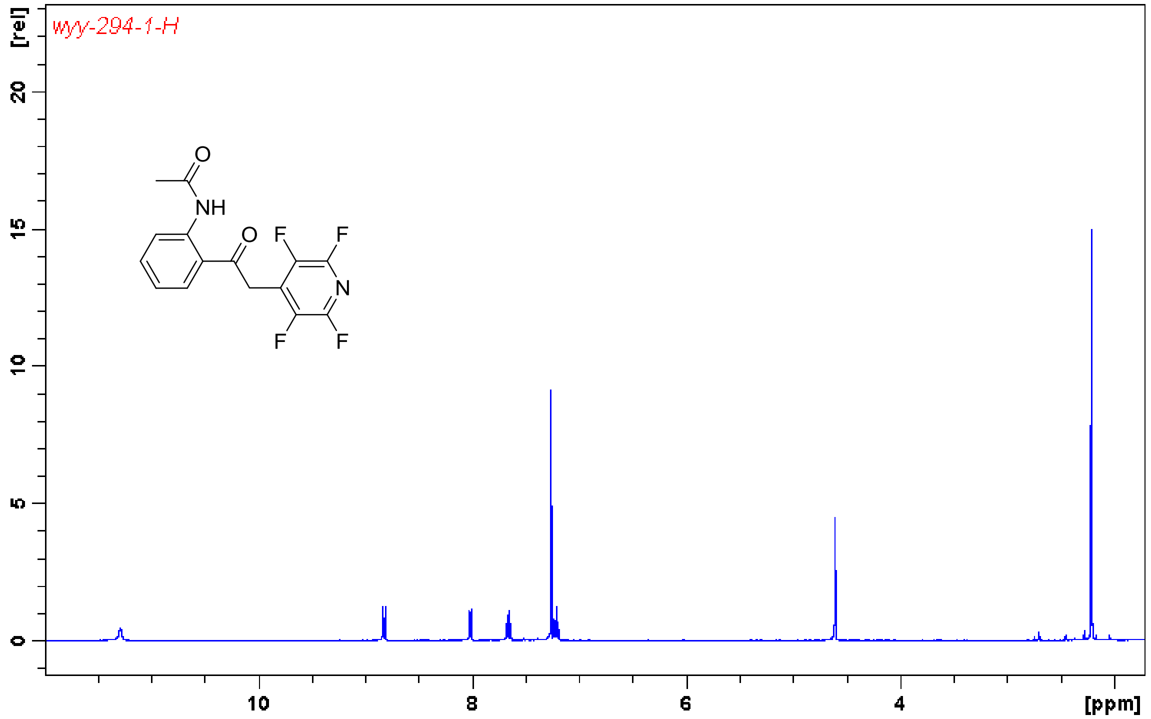
wyy-294-1-H
[rel]
20
15
10
5
0
O
NH
O
F
F
N
F
F
10
8
6
4
[ppm]

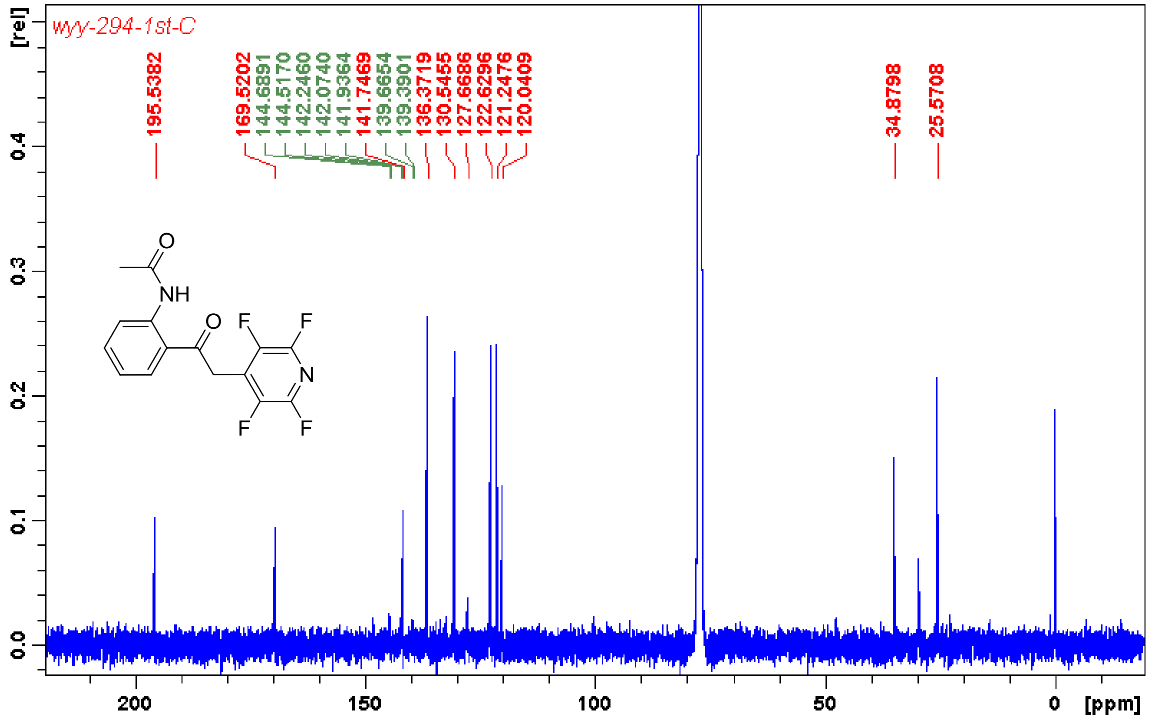
wyy-294-1st-C
[rel]
195.5382
169.5202
144.6891
144.5170
142.2460
142.0740
141.9364
141.7469
139.6654
139.3901
136.3719
130.5455
127.6686
122.6296
121.2476
120.0409
34.8798
25.5708
0.4
0.3
0.2
0.1
0.0
O
NH
O
F
F
N
F
F
200
150
100
50
0
[ppm]

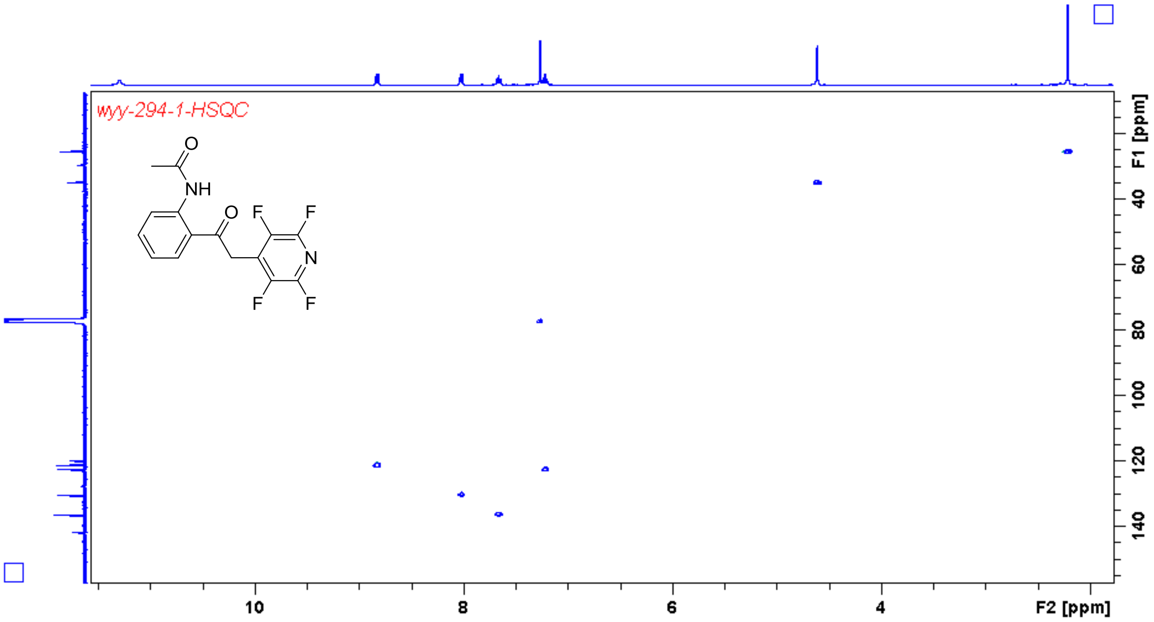
wyy-294-1-HSQC
O
NH
O
F
F
N
F
F
F1 [ppm]
40
60
80
100
120
140
10
8
6
4
F2 [ppm]

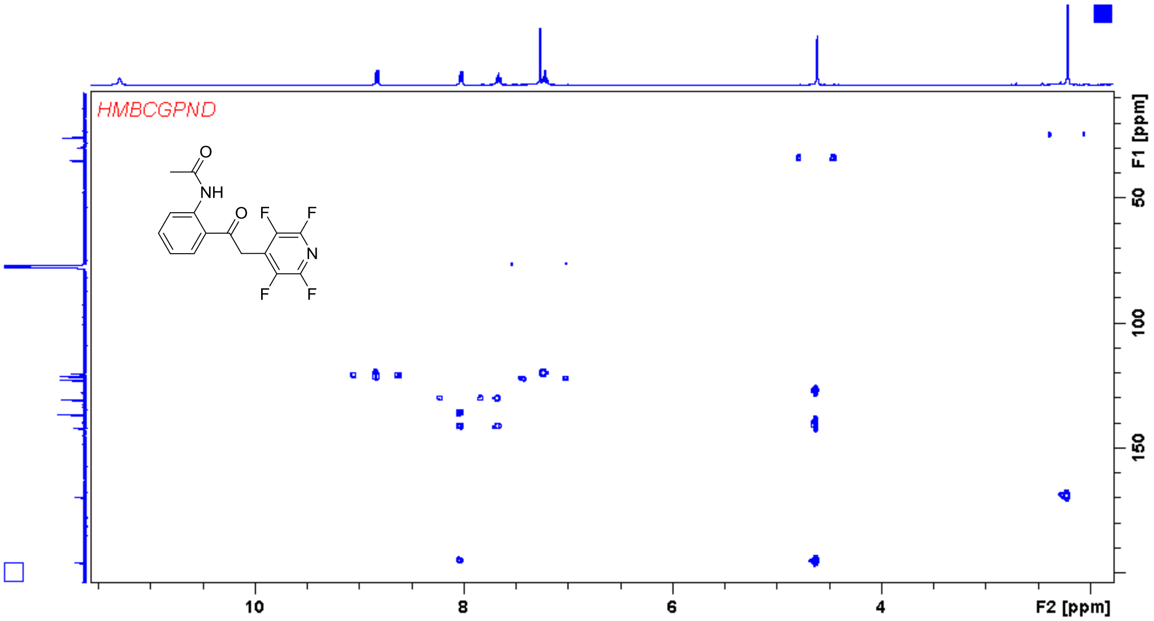
HMBCGPND
O
NH
O
F
F
N
F
F
F1 [ppm]
50
100
150
10
8
6
4
F2 [ppm]

References

1. Spencer, J.; Rathnam, R. P.; Patel, H.; Anjum, N. *Tetrahedron* **2008**, *64*, 10195-10200.

2. Yamane, Y.; Liu, X.; Hamasaki, A.; Ishida, T.; Haruta, M.; Yokoyama, T.; Tokunaga, M. *Org. Lett*. **2009**, *11*, 5162-5165.
